# Two-Country HANK and trade friction

**DOI:** 10.1371/journal.pone.0288976

**Published:** 2023-07-31

**Authors:** Yujie Yang, Chenxing Zhang, Wenwen Hou

**Affiliations:** 1 Jinhe Center for Economic Research, Xi’an Jiaotong University, Xi’an, China; 2 School of Aerospace Engineering, Xi’an Jiaotong University, Xi’an, China; University of Almeria, SPAIN

## Abstract

Compared to the Two-Country Representative Agents and the Small Open Economy Heterogeneous Agents models, this paper develops a Two-Country Heterogeneous Agents New Keynesian model, building a heterogeneous and endogenous channel of mutual investments on foreign illiquid assets and exploring two countries’ wealth distribution and inequality. This model explores the impacts of trade barriers on the two countries’ economic structures and behaviors. In the symmetric economy, the first launcher of tariffs suffers higher losses from the trade war, the other that does not launch tariffs suffers fewer losses. The two countries both suffer higher losses if the two sides simultaneously launch the trade war. However, in the asymmetrical economy, the country with a larger economic scale and higher productivities suffers lower losses from the trade war. Thus the high-tech country has the motivation to launch the trade war at a lower cost than the low-tech country. This paper implies that the trade war only occurs in asymmetric economies. The low-tech country can change its dilemma by improving technology because higher technology can enhance the quality level of products, stimulate economic consumption and investments, and finally offset the loss from tariff friction. Meanwhile, the trade detour is also an effective means to reduce the loss resulted from trade friction.

## 1 Introduction

Tax is an effective and direct tool for exploring the mechanism of two countries’ trade friction. Usually, in a trade war, the government wants to reduce consumption for imported goods by levying tariffs and restrict capital outflows by levying income tax for foreign investments. Changes in tax influence heterogeneous households’ consumptions and investments through the prices of imported goods and interest rates of foreign investments. If the home government levies tariffs on imported goods, the prices of imported goods will rise, and home households will consume less than before. Meanwhile, If the government restricts home households from investing in foreign assets by levying income tax, the home households will reduce investments in foreign assets. All of these changes will lead to heterogeneous households reallocating their assets. In the general equilibrium model, we first need to differentiate the impacts of tariffs on homogeneous and heterogeneous households’ consumption. Besides, we need to answer several questions: How do tariff shocks transmit between two countries in the Heterogeneous Agents(HA) and Representative Agents (RA) models? Compared to the Two-Country Representative Agents(Two-Country RA) model and Small Open Economy Heterogeneous Agents(SOE-HA) model, what are the new characteristics of the Two-Country Heterogeneous Agents(Two-Country HA) model?

This paper develops two transmitting channels on prices of imported goods and interest rates of foreign investments. Further, it creates a new bridge where two countries’ heterogeneous households can mutually invest in foreign illiquid assets. The Two-Country HA model assumes that heterogeneous households hold three kinds of assets for developing this bridge: bonds, home illiquid assets, and foreign illiquid assets.

The first transmitting channel of this paper is relative to the endogenous and heterogeneous consumption of imported goods, unlike the SOE-HA [1] and Two-Country RA models [2]. In these commonly used benchmark models, the changes in aggregated consumption are affected by the prices of imported goods. However, heterogeneous changes in consumption cannot be explored in the homogeneous model, and economic inequality is also unclear in the Two-Country RA model. Under the same marginal propensity to consume, confronting the positive shocks of prices on imported goods, will the poor and the wealthy respond consistently? The poor may reduce the imported goods only slightly because they usually consume little imported goods before. However, the wealthy may be the opposite. The sharp difference in tariffs on the imported goods between the Two-County RA and Two-County HA models lies in the impacts of tariffs of imported goods on the two countries’ wealth distribution. Therefore, we build the Two-Country Heterogeneous Agents(Two-Country HA) model to explore the changes in the wealth structure caused by the tariffs.

The second transmitting channel relates heterogeneous households’ foreign investments. In a real trade war, the government may restrict foreign investments and thereby prevent the opponent from expanding total demand. Unlike the classical Two-County RA model, this channel of foreign investments is heterogeneous. Home households use the home currency to invest in foreign assets. Therefore, there are two channels for capital flowing in the Two-Country HA model, consuming imported goods and investing in foreign assets. Why does this paper create this channel of mutual investments in foreign illiquid assets rather than in liquid assets? Outputs are the function of aggregated illiquid assets. Changes in investments in illiquid assets can be directly transmitted into the two countries’ outputs. In contrast, in the classical Two-Country RA model [2], changes in investments of liquid assets do not directly affect the two countries’ outputs and only transmit in the household sector.

Why is it essential to create this new channel of investments and explore the tax transmitting mechanism in the Two-Country HA model rather than the Two-Country RA model [2] There are two factors to explain this problem. On the one hand, in the Two-Country RA model, tariffs mainly transmit through two countries’ consumption and then affect other economic variables, such as bonds and illiquid assets. Although we can add this channel of investments on the foreign illiquid assets in the Two-Country RA model, confronting the same tax of investments on the foreign assets, heterogeneous households have distinguished behaviors. For example, raising the tariffs on foreign investments has little impact on the poor because they originally had small investments in foreign assets. However, the wealthy may reduce foreign investment to a large degree. These heterogeneous changes only can be shown in the Two-Country HA model; On the other hand, measuring inequality is important in the trade war. A significant finding in the Two-Country HA model is that tax impacts on the investments of foreign assets will be directly shown in the heterogeneous households’ wealth distribution. Therefore, in the Two-Country HA model, inequality of liquid and illiquid assets can be easily measured through Gini-coefficients.

Why does this paper study the transmitting mechanism of tariffs in the Two-Country HA model rather than the Small Open Economy HA model? First, the Small Open Economy HA model [1], can depict changes in wealth distribution after the tariffs shock, but the channel of consumption and foreign investments are exogenous. The SOE-HA model [3], lacks the endogenous channels of consumption and investment where home households can invest in foreign assets, and foreign households can invest in home assets. Therefore, the SOE-HA model’s tariffs and interest rate shocks only transmit in the home economy and cannot affect the foreign economy. However, in the Two-Country HA model, home shocks can transmit into foreign economic variables through consumption and investments on foreign assets, and thus we can explore two countries’ mutual economic behaviors. Second, the two countries’ policy cooperation and noncooperation can be clearly simulated in the Two-Country HA model. If one of these two countries firstly launches the tariffs, the other will strike back by the first launcher’s economic scale and structure. It isn’t easy to explore these mutual interactions in the SOE-HA model.

### 1.1 Literature review

Our article is mainly based on heterogeneity and the open economy. The heterogeneous literature focuses on the breakthrough of modeling technology and solution algorithm, while the research of open economy is more inclined to the description of macroeconomic operation mechanism.

Heterogeneous research mainly includes its origin and emerging breakthroughs. In terms of its origin, heterogeneity in the dynamic macroeconomic model was initially developed in the 1980s. The difficult point lies in aggregating heterogeneous households by the joint distribution function. Krusell and Smith [4, 5] overcome this problem and build a heterogeneous households model aggregated by the joint distribution, but its algorithm cannot solve the multi-dimensional steady state. However, the emerging heterogeneous models have made progress in both continuous and discrete directions and developed the corresponding algorithm to solve the steady states of multiple heterogeneous variables. In the continuous field, Kaplan et al. [6] take the assumption of heterogeneous agents into the New Keynesian framework, build a first continuous Heterogeneous Agents New Keynesian model, and develop the corresponding algorithm based on the Economics of Inaction. In the discrete field, Ottonello and Winberry [3] and Winberry [7] develop an algorithm to iterate the value function and solve the heterogeneous agents’ models, but the complete discrete framework is built by Bayer and Luetticke [8], Bayer et al. [9] and Auclert et al. [10, 11]. Bayer et al. [12] develop the Endogenous Grids Methods(EGM) algorithm to compute this model, which has a relatively perfect performance to solve the steady-state but high time cost to calculate this model. Unlike the EGM algorithm, Auclert et al. [10, 11] develop a Sequential Jacobian Method to compute and simulate the HANK model, which improves the computing efficiency of steady states and thus saves large amounts of computing time. Although these continuous and discrete heterogeneous models’ solution algorithms are distinguished, their results of steady states and simulations are similar. Our article mainly expanded the continuous framework in the Kaplan et al. [6] and developed the algorithm solving steady states in the two countries.

The study of the open economy is divided into two stages: the first is the classical homogeneous framework before introducing heterogeneity, and the second is the emerging framework after introducing heterogeneity. The former studies monetary interest rate transmission under a single-agent assumption, while the latter focuses on studying heterogeneous households’ wealth distribution. In the homogeneous framework, the benchmark model on the Two-Country is built by Galí and Monacelli [2], which emphasizes the transmission mechanism of interest rate and exchange rate in the international market; Then Auray et al. [13] develop an endogenous tariffs model and explore the impacts of trade protection through the Two-Country RANK model based on the Galí and Monacelli [2]. In the heterogeneous framework, researchers mainly focus on the impact of financial market fluctuations on the heterogeneous households’ wealth distribution by the SOE-HANK model. Auclert et al. [1] find that inducing the heterogeneity of households results in this phenomenon: rising import prices lowers households’ real income and leads households to reduce spending, which weakens monetary transmission. Guo et al. [14] explore the role that international financial integration plays in monetary transmission. Their work discloses the connections between the exchange rate regime, globalization, monetary shocks, and households’ wealth distribution. Zhou [15] researches the impacts of the exogenous shocks on the households’ aggregated consumptions and Marginal Propensity To Consume (MPC). This work factually characterizes the impacts of external shock on consumption and home households’ wealth redistribution. The common characteristics of all the emerging literature above are to explore the impacts of monetary policy on home households’ wealth redistribution through marginal propensity to consume, exchange rate regime, and asset portfolios; however, these impacts are exogenous. Unlike these researches before, our paper’s model explored the endogenous effects on the two countries’ variables.

Our paper is organized as follows: Section 1 describes the introduction. Section 2 introduces the difference between the Small Open HANK, Two-Country RA, and Two-Country HANK. Section 3 mainly introduces the benchmark model. Section 4 depicts the mechanism of two countries’ friction on trade. Section 5 describes the trade friction in the symmetric framework. Section 6 describes the trade friction in the asymmetric framework. Section 7 is an analysis of trade data on the US and China. Section 8 is about third-party countries. Section 9 is the conclusions.

## 2 Small Open Economy HA, Two-Country RA, and Two-Country HA

Differing from the Small Open Economy Heterogeneous Agents model and Two-Country Representative Agents model, the most prominent characteristics of the Two-Country Heterogeneous Agents model are the transmitting mechanism and households’ wealth distribution.

In terms of the transmitting mechanism, the Two-Country HA model develops two heterogeneous and endogenous channels on the consumption of imported goods and investment for foreign illiquid assets where two countries’ households can directly consume foreign goods and invest in each other’s illiquid assets. These consumption and investment behaviors will change households’ asset allocations. However, the Small Open Economy Heterogeneous Agents model lacks this endogenous investment channel, and the Two-Country RA model fails to depict heterogeneous changes of consumption and illiquid assets, lacking analysis of wealth distribution. Especially, the two-Country RA model only depicts the two countries’ capital flowing through imported goods and liquid assets. In fact, foreign investments for the illiquid assets also will affect the two countries’ outputs. As for the wealth distribution, the Two-Country Heterogeneous Agents model can deeply depict the impacts of tariffs and income tax on the two countries’ inequality which is impossible in the Two-Country HA and SOE-HA models.

### 2.1 Wealth distribution and Gini-coefficient

As this paper shows, the distribution function is *g*(*b*_*t*_, *a*_*ht*_, *a*_*ft*_, *z*_*t*_), where *a*_*ht*_, *a*_*ft*_, and *b*_*t*_ are the home illiquid assets, foreign illiquid assets and aggregated liquid assets held by home households.

$$\begin{array}{c} A_{ht}=\int a_{ht}dg,\;A_{ft}=\int a_{ft}dg,\;B_{t}=\int b_{t}dg \end{array}$$ (1)

This paper assumes that *g*_*i*,*ht*_ denotes the distribution function on the interval (0, 1) of *a*_*ht*_. All the values of home illiquid assets *a*_*ht*_ are sorted into series *a*_*i*,*ht*_, *i* ∈ (0, 1), satisfying $a_{i_{1},ht}<a_{i_{2},ht}$ for all *i*_1_ < *i*_2_, *i*_1_, *i*_2_ ∈ (0, 1), and so do the foreign illiquid assets *a*_*i*,*ft*_.

Then, the Gini coefficient of *a*_*h*,*t*_ is given as:

$$\begin{array}{c} Gini_{a_{ht}}=2\int_{0}^{1}(j-\int_{0}^{i}\frac{a_{i,ht}}{A_{ht}}di)dj \end{array}$$ (2)

### 2.2 Endogenous and heterogeneous channel of consumption for imported goods

Here, this paper assumes that the foreign country levies tariffs for the imported goods from the home country. Subsequently, these tariffs levied on the price of the foreign country’s imported goods, $P_{f}^{*}$, will transmit from the export into the domestic economy of the home country. Tariffs will make the price of imported goods of the foreign country fluctuate, $dp_{f}^{*}$. Then this paper next explores the transmitting mechanism of $dp_{f}^{*}$.

For the two countries, home country and foreign country, total consumption of home country **C**_**t**_ is consist of two parts: consumption on the home goods **C**_**ht**_ and consumption on the imported goods **C**_**ft**_. Total consumption of foreign country $C_{t}^{*}$ is consist of two parts: consumption on the home goods $C_{\text{ht}}^{*}$ and consumption on the imported goods $C_{\text{ft}}^{*}$.

From the market clearing conditions in the Two-Country HA model, we know that the consumption is the significant part of aggregated outputs, and we simplify the aggregated outputs as the $Y_{t}=C_{\text{ht}}+C_{\text{ft}}^{*}$. Thus, the outputs and consumption are both also affected by the price of imported goods of the foreign country, that is, the consumption of the home country is the function on the price of imported goods of the foreign country, $Y_{t}\left(P_{f}^{*}\right)=C_{\text{ht}}\left(P_{f}^{*}\right)+C_{\text{ft}}^{*}\left(P_{f}^{*}\right)$. Here, this paper assumes that the foreign country government levies the tariffs on the imported goods and makes positive shocks on the price of imported goods.

**Proposition 1** *In response to the shock on the price of the foreign country’s imported goods, the impulse response of consumption is given by*

$$\begin{array}{c} \underset{\text{Channel}\;\text{of}\;\text{tariffs}\;\text{transmitting}\;}{\underset{︸}{\frac{dY}{dp_{f}^{*}}}}=\underset{\;\text{SOE-HA,}\;\text{Two-Country}\;\text{HA,}\;\text{Two-Country}\;\text{RA}\;\;}{\underset{︸}{\frac{dC_{h}}{dp_{f}^{*}}+\frac{dC_{f}^{*}}{dp_{f}^{*}}}} \end{array}$$ (3)

*but*

$$\begin{array}{c} \underset{\text{SOE-HA}\;}{\underset{︸}{\frac{dY}{dp_{f}^{*}}}}\neq \underset{\text{Two-Country}\;\text{HA}\;}{\underset{︸}{\frac{dY}{dp_{f}^{*}}}}\neq \underset{\text{Two-Country}\;\text{RA}\;}{\underset{︸}{\frac{dY}{dp_{f}^{*}}}} \end{array}$$ (4)

Usually, domestic exports will reduce if the foreign country raises tariffs for the imported goods. Therefore, $dp_{f}^{*}>0$ will make the home county’s exported goods $C_{\text{ft}}^{*}$ (the foreign country’s imported goods) decrease. Finally, these changes of exported goods affect the home total outputs and then transmit into the whole economy.

Proposition 1 indicates that the tariffs’ transmitting mechanisms in the SOE-HA, Two-Country RA, and Two-Country HA models are different. This transmitting mechanism in the SOE-HA seems similar to that in the Two-Country HA model. In the open Heterogeneous Agents model, households consumption for the imported goods is heterogeneous. However, there is a key difference. The imported goods are exogenous in the SOE-HA model, while the consumption for imported goods $C_{f}^{*}$ is endogenous in the Two-Country HA model. Differing from the SOE-HA, in the Two-Country HA model, foreign households also consume goods produced in the home. Therefore, two countries’ households have interactions on consumption goods, which will affect their balance sheets and utilities.

Compared to the Two Country RA model, heterogeneity does not change the transmitting channel of tariffs on the price of imported goods in the Representative Agents model. However, although households have the same preferences of consumption, the economic actions of households with different wealth distribution are distinguished. Thus, heterogeneous households’ wealth distribution will be changed, and measuring the inequality after tariffs shock is essential.

### 2.3 Endogenous and heterogeneous channel of capital flowing

The aggregated illiquid assets of home country, **K**_**t**_, consists of two parts, investments on the home illiquid assets **A**_**ht**_ and foreign investments on the home illiquid assets $A_{\text{ft}}^{*}$, thus $K_{t}=A_{\text{ht}}+A_{\text{ft}}^{*}$. Similarly, the aggregated illiquid assets of the foreign country is given by $K_{t}^{*}=A_{\text{ht}}^{*}+A_{\text{ft}}$. Here this paper linearizes the function of outputs, **Y** = *α***K** + (1 − *α*)**N** + **Z**. **K** is the aggregated illiquid assets, **N** is the aggregated labor supply and **Z** is the technology level. Capital **K** is the function of the interest rate **r**_**af**_. Thus, we simplify it as **Y**(**r**_**af**_) = **K**(**r**_**af**_).

**Proposition 2** *Differing from the SOE-RA, SOE-HA, and Two-Country RA model, there is a new channel for capital flowing between two countries,*

$$\begin{array}{c} \frac{dY}{dr_{\text{af}}}=\frac{dK}{dr_{\text{af}}},\;\;\;\;\frac{dY^{*}}{dr_{\text{af}}}=\frac{dK^{*}}{dr_{\text{af}}} \end{array}$$ (5)

*and*

$$\begin{array}{c} \frac{dK}{dr_{\text{af}}}=\underset{\text{Two-Country}\;\text{RA,}\;\text{SOE-HA}\;}{\underset{︸}{\frac{dA_{h}}{dr_{\text{af}}}}}+\underset{\text{Two-Country}\;\text{HA}\;}{\underset{︸}{\frac{dA_{f}^{*}}{dr_{\text{af}}};}}\;\;\underset{\text{Two-Country-HA}\;}{\underset{︸}{\frac{dK^{*}}{dr_{\text{af}}}}}=\frac{dA_{h}^{*}}{dr_{\text{af}}}+\frac{dA_{f}}{dr_{\text{af}}} \end{array}$$ (6)

In the classical Two-Country RA model, two countries are connected by the consumption goods rather than the mutual investments, while in the Two-Country HA model, there is an extra channel for capital flowing, foreign households can invest in home assets, and foreign currencies also flow into the home country. We can also develop this channel for capital flowing on liquid assets in the Two-Country RA model. However, the capital flowing in the liquid assets fails to transmit into the production function directly. Besides, the Two-Country RA cannot explore heterogeneous households’ changes of wealth. However, in the Two-Country HA model, the shock to the interest rate can be transmitted into the home, and heterogeneous foreign households finally affect households reallocating their portfolios.

In the SOE-HA model, the flowing channel of capital is exogenous. Households in the foreign country cannot invest the illiquid assets in the home country. Thus, *d* **r**_**af**_ has no impacts on the K^*^. The classical SOE-RA models cannot develop the endogenous channel of capital flowing and fail to measure heterogeneous changes of household portfolios.

### 2.4 SOE-HA and Two-Country HA

**Fig 1:**
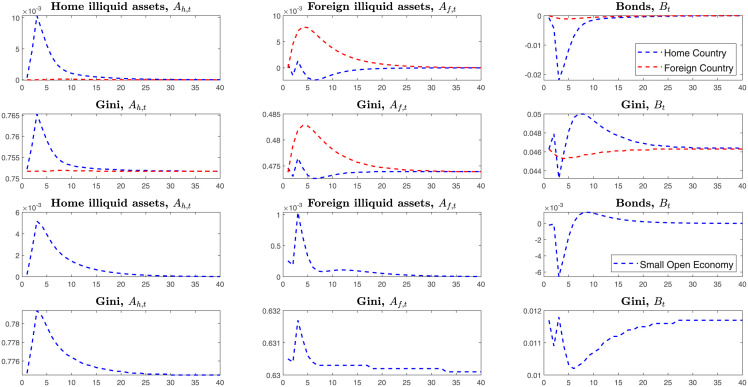
IRFS under the tariffs shock on the Small Open and Two-Country HANK.

This section simulates the tariffs shock in the SOE-HA and Two-Country HA models. This paper assumes that the home country levies tariffs on imported goods from the foreign country. Compared with the Small Open Economy, the Two-Country HANK model has a different transmitting channel where tariffs will affect the foreign country’s endogenous variables. Thus, we can explore the foreign country’s economic behaviors in the Two-Country HA model. In the SOE-HA model, the home country cannot detect the foreign country’s economic behaviors. From the Fig 1 above, we can find that the Gini coefficient in the SOE-HA model is higher than the Two-Country-HA model. That is, the endogenous channel of capital outflows makes the Gini-coefficient decrease and reduces the wealth inequality.

Unlike the SOE-HA and Two-Country HA model, the Two-Country RA model cannot explore heterogeneous households’ wealth distribution. From the Fig 1, we can find the Gini-coefficients become higher after launching tariffs shock on the imported goods, which means that the country’s inequality deteriorates.

## 3 Model

This section characterizes the Two-Country HANK model. Incorporating the Kaplan et al. [6] and Galí and Monacelli [2], we develop the algorithm of the Hamilton-Jacobi-Bellman equation containing the four heterogeneous variables and build the heterogeneous Two-Country model. Two countries’ economic behaviors can be explored in this model when confronted the trade friction. This paper assumes there are two countries, the home and foreign country, with similar structures. For the assumption of sticky price, we follow the form Kaplan et al. [6]. Unlike the small open HANK model in the De Ferra et al. [16], all the main variables of the foreign country are endogenous in this Two Country HANK model.

There is a complete space with four dimensions, $\Omega =Z\times B\times K_{h}\times K_{f}$, which consists of heterogeneous households’ productivities *z*_*i*_, $z_{i}\in Z$, liquid assets *b*_*it*_, $b_{it}\in B$, home illiquid assets *a*_*h*,*it*_, $a_{h,it}\in K_{h}$, and households investments for the foreign country’ illiquid assets, *a*_*f*,*it*_, $a_{f,it}\in K_{f}$. Heterogeneous households with various portfolios are contained and converged in this space, and the *g*_*t*_ is the home country’s joint distribution function. As the home country, the foreign country’s space is the $\Omega^{*}=Z^{*}\times B^{*}\times K_{h}^{*}\times K_{f}^{*}$. $g_{t}^{*}$ is the foreign country’s joint distribution function.

### 3.1 Households

We build the three assets heterogeneous model under the continuous framework where the time is continuous, and the horizon is infinite. The two countries’ structures are similar, and thus we choose one of them to describe its economy. For the home country, heterogeneous households’ preference follows the form of CRRA utility function, as

$$\begin{array}{c} u(c_{ht},c_{ft})=\frac{c_{ht}{}^{1-\gamma_{c}}}{1-\gamma_{c}}+\varphi_{c}\frac{c_{ft}{}^{1-\gamma_{c}}}{1-\gamma_{c}}-\varphi_{l}\frac{\ell_{t}^{1+\gamma_{\ell }}}{1+\gamma_{\ell }} \end{array}$$ (7)

Heterogeneous households consume two kind of goods, *c*_*ht*_ and *c*_*ft*_. *c*_*ft*_ denotes consumption of the imported goods from the foreign country. *c*_*ht*_ is the consumption of home goods. *φ*_*c*_ is the exogenous parameter, measuring the weight of imported goods in the utility function. *φ*_*l*_ is the exogenous parameter on the labor, denoting the weight of labor in the utility function. *γ*_*c*_ is the risk aversion coefficients, and *γ*_*l*_ is the reciprocal of Frisch elasticity.

The demand of consumption is directly decided by its price. For the price system of domestic and imported foreign goods, the prices for the two kinds of goods are *P*_*ht*_ and *P*_*ft*_*e*_*t*_(1 + *τ*_*im*_), respectively. Assume that *P*_*t*_ denotes the total price level of the home country and $P_{t}^{*}$ denotes the total price level of the foreign country. *P*_*ht*_ and *P*_*ft*_ are prices of domestic and imported foreign goods. *τ*_*im*_ is the tariffs for the imported goods. *e*_*t*_ is the exchange rate between the home and foreign country. Home households purchase foreign goods and pay prices at the exchange rate *e*_*t*_. Thus, the real prices, purchasing the domestic goods and imported goods are the $\frac{P_{ht}}{P_{t}}$ and $\frac{P_{ft}e_{t}\left(1+\tau_{im}\right)}{P_{t}}$, respectively. In the HANK model, households’ productivities, *z*_*t*_, are also heterogeneous, which follows a stochastic process in Kaplan et al. [6]. In each country, the economy is populated by heterogeneous continuous households. Households take the CRRA utility functions. In the HANK model, households take the wage and interest rate of illiquid assets as given, where the wage and interest rate are the results of optimization in the firms’ sector.

Households will choose consumption and portfolio to maximize their utility:

$$\begin{array}{c} E_{0}\int_{0}^{\infty }e^{-\rho t}u(c_{ht},c_{ft},\ell_{t})dt \end{array}$$ (8)

The income constraint is given as:

$$\begin{array}{c} \begin{array}{cc} {\dot{b}}_{t}= & (1-\tau_{w})w_{t}z_{t}\ell_{t}+r_{b}b_{t}+T_{t}-d_{ht}-\chi_{h}-d_{ft}\\ & -\chi_{f}-c_{ht}\frac{P_{ht}}{P_{t}}-c_{ft}\frac{P_{ft}e_{t}\left(1+\tau_{im}\right)}{P_{t}}\\ {\dot{a}}_{ht}= & r_{ah}a_{ht}+d_{ht}\\ {\dot{a}}_{ft}= & r_{af}a_{ft}+d_{ft}\frac{P_{t}}{P_{t}^{*}e_{t}},\;b_{ht}\geq \underline{b}\;a_{ht},a_{ft}\geq 0. \end{array} \end{array}$$ (9)

Where *ρ* denotes the households discount rate, and *ℓ*_*t*_ is the labor supply. Heterogeneous households hold three assets: liquid assets *b*_*t*_, domestic illiquid assets *a*_*ht*_, and foreign illiquid assets *a*_*ft*_. *τ*_*w*_ is the tax rate of wage, *r*_*b*_ is the real interest rate of liquid assets. *r*_*ah*_ is the interest rate of home illiquid assets. *r*_*af*_ is the interest rate of foreign illiquid assets. Note, *r*_*ah*_ = *r*_*k*_ − *δ*, *r*_*k*_ is the interest rate that firms pay to households. *δ* is the depreciation rate. In this model, savings in liquid assets ${\dot{b}}_{t}$ is equal to household’s income stream, which is composed of net wage (1 − *τ*_*w*_)*w*_*t*_*z*_*t*_*ℓ*_*t*_, government transfer *T*_*t*_, net of deposits into or withdrawals from the home illiquid account *d*_*ht*_ and foreign illiquid account *d*_*ft*_, transaction costs *χ*_*h*_ and *χ*_*f*_, home consumption *c*_*ht*_, and foreign consumption *c*_*ft*_. Net savings in illiquid assets ${\dot{a}}_{ht}$ is equal to interest payments on home illiquid assets plus net deposits from the liquid account *d*_*ht*_; Net savings in illiquid assets ${\dot{a}}_{ft}$ is equal to interest payments on foreign illiquid assets plus net deposits from the liquid account *d*_*ft*_. Households can borrow from the market with interest rate *r*_*b*_ + *κ*, by paying an extra interest rate gap *κ*. Then, the total borrowing cost *κ*∫_Ω_ max(*b*_*t*_, 0)*dg*_*t*_.

### 3.2 Firms

#### 3.2.1 Final goods firm

There is a final firm in the economy, producing the total outputs *Y*_*t*_ by using all kinds of intermediate goods *y*_*jt*_, *j* ∈ (0, 1),

$$\begin{array}{c} Y_{t}={\left(\int_{0}^{1}y_{jt}^{\frac{\varepsilon -1}{\epsilon }}dj\right)}^{\frac{\varepsilon }{\varepsilon -1}} \end{array}$$ (10)

Note that this paper follows the assumption in the Kaplan et al. [6] and assumes that all the intermediate firms are homogeneous. *ε* > 0 is the elasticity of substitution across various intermediate goods.

The cost minimized problem is,

$$\begin{array}{c} \begin{array}{cc} \underset{y_{jt}}{\text{min}\;}\; & Y_{t}P_{ht}-\int_{0}^{1}y_{jt}p_{jt}dj\\ st\; & Y_{t}={\left(\int_{0}^{1}y_{jt}^{\frac{\varepsilon -1}{\epsilon }}dj\right)}^{\frac{\varepsilon }{\varepsilon -1}} \end{array} \end{array}$$ (11)

from the cost minimization, we have the final goods’ demand for intermediate goods,

$$\begin{array}{c} y_{j,t}={\left(\frac{p_{jt}}{P_{ht}}\right)}^{-\varepsilon }Y_{t},\;\text{where}\;P_{ht}={\left(\int_{0}^{1}p_{jt}^{\left(1-\varepsilon \right)}dj\right)}^{\frac{1}{1-\varepsilon }} \end{array}$$ (12)

#### 3.2.2 Intermediate goods firms

There is a continuum of homogeneous intermediate firms indexed by *j* ∈ [0, 1], which produce intermediate goods *y*_*jt*_. Intermediate firms have same total factor productivity level *Z*_*t*_, and same Cobb-Douglas product function:

$$\begin{array}{c} y_{jt}=Z_{t}k^{\alpha }l_{jt}^{\left(1-\alpha \right)}, \end{array}$$ (13)

where *α* ∈ (0, 1) is the share of capital. Intermediate goods firms are monopolistically competitive, using effective labor *l*_*jt*_ and effective capital *k*_*jt*_ to produce intermediate goods *y*_*jt*_.

Intermediate goods firms hire labor at wage *w*_*t*_ in the competitive market and rent capital at return rate *r*_*k*_ in the competitive market.

Marginal cost *mc*_*t*_ is derived from the cost minimization,

$$\begin{array}{c} mc_{t}=\frac{1}{Z_{t}}{\left(\frac{r_{kt}}{\alpha }\right)}^{\alpha }{\left(\frac{w_{t}}{1-\alpha }\right)}^{1-\alpha } \end{array}$$ (14)

According to Kaplan et al. [6], this paper follows the assumption of sticky price in Rotemberg [17],

$$\begin{array}{c} \Theta_{t}(\frac{{\dot{p}}_{t}}{p_{t}})=\frac{\theta }{2}{\left(\frac{{\dot{p}}_{t}}{p_{t}}\right)}^{2}Y_{t}, \end{array}$$ (15)

where the *θ* > 0 denotes coefficient of price adjustment cost.

Intermediate goods firms adjust goods prices *p*_*j*,*t*_ to maximize their profits,

$$\begin{array}{cc} \underset{p_{jt}}{\text{max}\;}: & \int_{0}^{\infty }e^{-\int_{0}^{t}r_{s}^{k}ds}\{\frac{p_{jt}}{P_{ht}}y_{jt}-mc_{t}y_{jt}-\frac{\theta }{2}{\left(\frac{{\dot{p}}_{jt}}{p_{jt}}\right)}^{2}Y_{t}\}dt\\ =\int_{0}^{\infty } & e^{-\int_{0}^{t}r_{s}^{k}ds}\{[(\frac{p_{jt}}{P_{ht}}-mc_{t}){\left(\frac{p_{jt}}{P_{h,t}}\right)}^{-\varepsilon }-\frac{\theta }{2}{\left(\frac{{\dot{p}}_{jt}}{p_{jt}}\right)}^{2}]Y_{t}\}dt, \end{array}$$

and have the New Keynesian Phillips Curve, which is also the same as Kaplan et al. [6]:

$$\begin{array}{c} (r_{kt}-\frac{{\dot{Y}}_{t}}{Y_{t}})\pi_{t}=\frac{1}{\theta }\left(-\left(1-mc_{t}\right)\varepsilon +1\right)+{\dot{\pi }}_{t}, \end{array}$$ (16)

where *π*_*t*_ is the inflation rate on the price of domestic goods *P*_*ht*_. (Derivation process in Appendix 10.1).

### 3.3 Clearing conditions on two countries

The clearing conditions of the two countries mainly contain the consumption, price system, liquid assets, illiquid assets, outputs, and government expenditure. This paper assumes that foreign households can invest in the home illiquid assets. The *g*_*t*_ and $g_{t}^{*}$ are the joint distribution functions on the two countries’ heterogeneous households. Thus we aggregate heterogeneous variables by these two distribution functions *g*_*t*_ and $g_{t}^{*}$. Here the total illiquid assets of home country consist of two parts: home households’ investments in the home illiquid assets *A*_*ht*_ and foreign households’ investments in the home illiquid assets $A_{ft}^{*}$. Hence, $A_{ht}=\int_{\Omega }^{}a_{ht}dg_{t}$ and $A_{ft}^{*}=\int_{\Omega^{*}}^{}a_{ft}^{*}dg_{t}^{*}$. That is, $K_{t}=A_{ht}+A_{ft}^{*}$.

$$\begin{array}{c} K_{t}=\int_{\Omega }^{}a_{ht}dg_{t}+\int_{\Omega^{*}}^{}a_{ft}^{*}dg_{t}^{*}. \end{array}$$ (17)

Foreign country’s clearing condition is similar to home country as the equation above. That is, foreign country’s total illiquid assets equal foreign country’s households’ investments in the foreign illiquid assets $a_{ht}^{*}$ and home country’s households’ investments in the foreign country’s illiquid assets *a*_*ft*_. $A_{ft}=\int_{\Omega }^{}a_{ft}dg_{t}$ and $A_{ht}^{*}=\int_{\Omega^{*}}^{}a_{ht}^{*}dg_{t}^{*}$, thus $K_{t}^{*}=A_{ht}^{*}+A_{ft}$.

$$\begin{array}{c} K_{t}^{*}=\int_{\Omega^{*}}^{}a_{ht}^{*}dg_{t}^{*}+\int_{\Omega }^{}a_{ft}dg_{t}. \end{array}$$ (18)

Where the integration aggregates the illiquid assets over the distribution function, *g*_*t*_ and $g_{t}^{*}$, in the space Ω and Ω*. All the interactions between these two countries are realized by this route in the illiquid assets market. All the shocks, such as the tax on the imported goods and the income of foreign illiquid assets, are linked and transmitted directly or indirectly by this channel.

Two countries’ consumptions are cleared as follows,

$$\begin{array}{c} C_{ht}=\int_{\Omega }^{}c_{ht}dg_{t},\;C_{ft}=\int_{\Omega }^{}c_{ft}dg_{t},\;C_{ht}^{*}=\int_{\Omega^{*}}^{}c_{ht}^{*}dg_{t}^{*},\;C_{ft}^{*}=\int_{\Omega^{*}}^{}c_{ft}^{*}dg_{t}^{*}. \end{array}$$ (19)

For the home country, the aggregated consumption consists of two parts, domestic consumptions *C*_*ht*_ and imported goods *C*_*ft*_. Similarly, the foreign country’s consumption also contains two parts, $C_{ht}^{*}$ and $C_{ft}^{*}$.

The liquid assets are aggregated by the following equations,

$$\begin{array}{c} B_{t}=\int_{\Omega }^{}b_{t}dg_{t},\;B_{t}^{*}=\int_{\Omega^{*}}^{}b_{t}^{*}dg_{t}^{*},\;B_{g}=B_{t}+Y_{t},\;B_{g}^{*}=B_{t}^{*}+Y_{t}^{*}. \end{array}$$ (20)

*B*_*g*_ and $B_{g}^{*}$ are the bonds that two countries’ governments issued, which means that government-issued bonds consist of liquid assets held by households and outputs.

Aggregated labor *L*_*t*_ and $L_{t}^{*}$, in the Cobb-Douglas production function, are cleared by the joint functions as follows,

$$\begin{array}{c} L_{t}=\int_{\Omega }^{}z_{t}\ell_{t}dg_{t},\;L_{t}^{*}=\int_{\Omega^{*}}^{}z_{t}^{*}\ell_{t}^{*}dg_{t}^{*}. \end{array}$$ (21)

The government’s expenditures are composed of the households’ income of government bonds and transfer payments in each country,

$$\begin{array}{c} \begin{array}{ccc} G_{t} & =B_{g}r_{b}+T_{t},\;G_{t}^{*} & =B_{g}^{*}r_{b}^{*}+T_{t}^{*}. \end{array} \end{array}$$ (22)

Goods markets clear when:

$$\begin{array}{c} \begin{array}{cc} Y_{t} & =C_{ht}+C_{ft}^{*}+I_{t}+G_{t}+\Theta_{t}+\chi_{h}+\chi_{f}+\kappa \int_{\Omega }\text{max}\;\left(b_{t},0\right)dg_{t}\\ Y_{t}^{*} & =C_{ht}^{*}+C_{ft}+I_{t}^{*}+G_{t}^{*}+\Theta_{t}^{*}+\chi_{h}^{*}+\chi_{f}^{*}+\kappa^{*}\int_{\Omega^{*}}\text{max}\;\left(b_{t}^{*},0\right)dg_{t}^{*} \end{array} \end{array}$$ (23)

The home country’s clearing condition is that outputs *Y*_*t*_ equal the sum of consumption *C*_*h*_, $C_{f}^{*}$, investment *I*, government spending *G*, transaction costs *χ*_*h*_, *χ*_*f*_, adjustment costs Θ, and households’ borrowing cost *κ*∫_Ω_ max(*b*_*t*_, 0)*dg*_*t*_.

This paper assumes that single heterogeneous household’s consumption satisfy,

$$\begin{array}{c} P_{t}c_{t}=P_{ht}c_{ht}+e_{t}P_{ft}\left(1+\tau_{im}\right)c_{ft}. \end{array}$$ (24)

The total price level *P*_*t*_ is determined by:

$$\begin{array}{c} P_{t}={\left[\rho_{p}P_{h,t}^{\varepsilon_{p}}+(1-\rho_{p}){\left(P_{f,t}e_{t}\left(1+\tau_{im}\right)\right)}^{\varepsilon_{p}}\right]}^{\frac{1}{\varepsilon_{p}}} \end{array}$$ (25)

Where *ε*_*p*_ denotes the substitution between domestic goods price *P*_*ht*_ and foreign goods price *P*_*ft*_, and *ρ*_*p*_ is the fraction of domestic goods price.

The total inflation rate is the change rate of the total price:

$$\begin{array}{c} \pi_{t}^{total}=\frac{\dot{P_{t}}}{P_{t}}. \end{array}$$ (26)

The Bank obeys the Taylor’s rule to adjust the nominal interest rate *i*_*t*_:

$$\begin{array}{c} \begin{array}{cc} i_{t} & =i_{s}+\rho_{\pi }\pi_{t}^{total}+\epsilon_{i}, \end{array} \end{array}$$ (27)

where *i*_*s*_ denotes the target nominal interest level, *ρ*_*π*_ is the inflation coefficient, and *ϵ*_*i*_ ∼ *N*(*μ*, *σ*_*i*_) is the exogenous shock variable. Relations of the nominal interest rate *i*_*t*_ and the real interest rate *r*_*b*_ follow $r_{b}=i_{t}-\pi_{t}^{total}$ (Fisher Equation).

The government’s budget constraint equation is:

$$\begin{array}{c} \begin{array}{cc} \tau_{im}C_{ft}\frac{P_{ft}e_{t}\left(1+\tau_{im}\right)}{P_{t}} & +\frac{\tau_{f}}{1-\tau_{f}}r_{af}A_{ft}\frac{P_{t}^{*}e_{t}}{P_{t}}+\tau_{w}w_{t}L_{t}=(B_{t}+Y_{t})r_{b}+T_{t}\equiv G_{t} \end{array} \end{array}$$ (28)

The left side is the government’s income items, including tariffs income on imported goods, income tax on the investments for foreign illiquid assets, and income of wage tax; The right side is government expenditures.

### 3.4 Equilibrium

The algorithm solving the Two-Country HANK model originally comes from the book “Economics of Inaction” by Stokey [18] and Kaplan et al. [6]. The kernel of this algorithm is to solve the two HJB equations on the two countries and the clearing conditions. Unlike the emerging literature on the open economy HANK, it is more difficult to let two HJB equations with four heterogeneous variables simultaneously converge in the closed-loop in the Two-Country HANK.

**Definition 1** *Given the tax shocks* $\left\{\tau_{im,t},\tau_{im,t}^{*},\tau_{if,t},\tau_{if,t}^{*}\right\}$ , *the processes for* $\left\{z_{it},z_{it}^{*}\right\}$, *prices* $\left\{w_{t},w_{t}^{*},r_{b,it},r_{b,it}^{*},r_{ah,it},r_{af,it},r_{ah,it}^{*},r_{af,it}^{*},P_{ht},P_{ft},P_{ht}^{*},P_{ft}^{*},P_{t},P_{t}^{*},e_{t},e_{t}^{*}\right\}$, *government expenditures* $\left\{G_{t},G_{t}^{*}\right\}$, *heterogeneous bonds’ holdings of two countries* $\left\{b_{it},b_{it}^{*}\right\}$, *heterogeneous illiquid assets’ holdings on the two countries* $\left\{a_{h,it},a_{f,it}^{*},a_{h,it}^{*},a_{f,it}\right\}$, *outputs* $\left\{Y_{t},Y_{t}^{*}\right\}$, *policy functions* $\left\{c_{it},c_{it}^{*}\right\}$ *and joint distribution functions* $\left\{g_{t},g_{t}^{*}\right\}$, *such that*

- *the factor returns rates are derived through optimizing the profits function of the firm.*
- *the steady states of assets,* $\left\{a_{h,it},a_{f,it}^{*},a_{f,it},a_{h,it}^{*},b_{it},b_{it}^{*}\right\}$ *, are solved by the loop on the two HJB equations on the home country and foreign country*.
- *the aggregated endogenous variables* $\left\{K,K^{*},C_{h},C_{h}^{*},C_{f},C_{f}^{*},B,B^{*},Y,Y^{*},L,L^{*}\right\}$ *are converged by the joint distribution functions* $\left\{g_{t},g_{t}^{*}\right\}$ .

**Fig 2:**
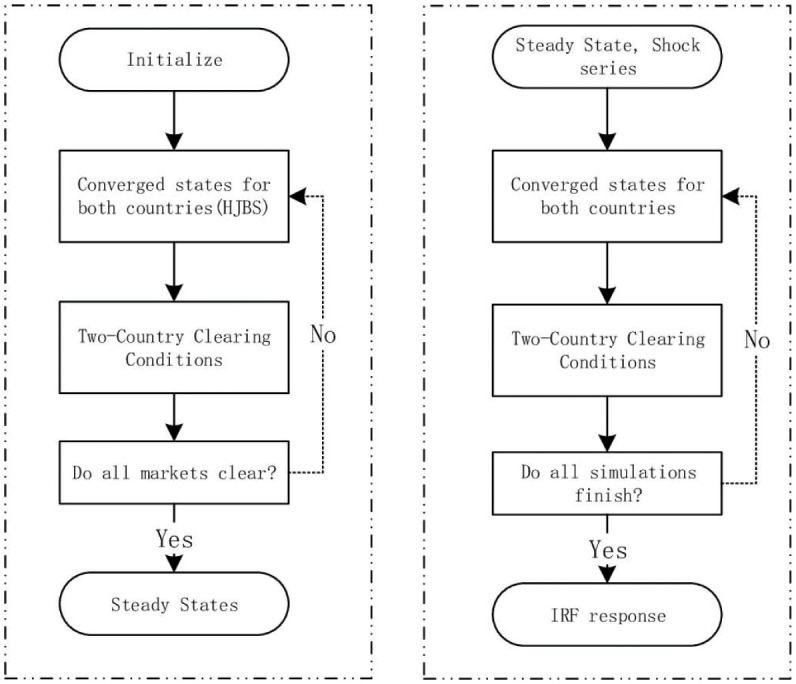
Solving steps for Two-Country HANK model.

The Fig 2 shows the processes of solving steady states and simulating shocks to the model, which mainly consists of solving the HJB equations.

#### 3.4.1 Steady state

Unlike the RANK model, in the HANK model, the kernel of solving steady states lies in the two HJB equations and KF equations, which are carefully introduced in Appendix 10.1. Here we introduce the steps in the logic figure above.

- Initialize the model and set all parameters and variables.
- Solve these two HJB equations and find the converged states for both countries.
- Check whether variables satisfy the clearing condition on the two countries.
- The loop ends if all market-clearing conditions are satisfied and all variables are converged. If not, go back to step 2 and iterate until variables are converged.

#### 3.4.2 Simulations and impulse responses

After having the steady states, we simulate the model through tariffs shocks, which mainly consist of two kinds of shocks: one of two countries launches tariffs shock, and two countries simultaneously launch tariffs shocks with each other.

- Initialize the model, set all parameters and variables with steady-state values, and load the shocks.
- Find the converged states for both countries under tariffs shocks.
- Update the foreign assets of each country using another country’s results in step 2.
- Check the end condition, record the endogenous variables in both countries, and load the next period shock values. Once all shock series are simulated, the loop ends. Otherwise, go to step 2 and continue the iteration.

### 3.5 Calibrations of parameters

We calibrate the model by all the parameters in the Table 1, mainly following Den Haan [19], Kaplan et al. [6], and Bayer et al. [9]. In the environment that two countries are symmetric, most of the parameters are the same because this paper assumes that the two countries have the same economic scales and structures. However, in the environment that two countries are asymmetric, some parameters on the two countries are different, especially for two countries’ productivities and amount of aggregated illiquid assets. The productivity of the home country is 3, and the foreign country is 5, (*Z*, Z*) = (3, 5).

**Table 1:** Parameters of model.

Parameter	Value	Description
**Households**		
*ρ*, ρ*	(0.9, 0.9)	Discount factor
$\gamma_{c},\gamma_{c}^{*}$	(2, 2)	Relative risk aversion coefficients
$\gamma_{\ell },\gamma_{\ell }^{*}$	(1, 1)	Elasticity of labor
$\varphi_{c},\varphi_{c}^{*}$	(2, 2)	Coefficients for imported goods
$\varphi_{\ell },\varphi_{\ell }^{*}$	(1, 1)	Coefficients for labor
$\tau_{w},\tau_{w}^{*}$	(0.1, 0.1)	Tax rate of wage
*e*, e*	(1, 1)	Exchange rate
*κ*, κ*	(0.5, 0.5)	Interest rate gap
**Production**		
*Z*, Z*	(3, 5)	Total factor productivity
*α*, α*	(0.33, 0.33)	Share of illiquid assets
*δ*, δ*	(0.025, 0.025)	Depreciation rate
*ϵ*, ϵ*	(10, 10)	Substitution elasticity
*θ*, θ*	(100, 100)	Coefficient on sticky price
$\in_{p},\in_{p}^{*}$	(0.5, 0.5)	Substitution elasticity on the price
$\rho_{p},\rho_{p}^{*}$	(0.9, 0.9)	Share of *P*_*ht*_
**Tariffs shock**		
$\rho_{\tau_{im}},\rho_{\tau_{im}}^{*}$	(0.86, 0.86)	Smooth coefficients
$\tau_{im,ss},\tau_{im,ss}^{*}$	(0.2, 0.2)	Tariffs at the steady states
$\mu_{\tau_{i}m},\mu_{\tau_{i}m}^{*}$	(0.5, 0.5)	Shocks’ means
$\sigma_{\tau_{i}m},\sigma_{\tau_{i}m}^{*}$	(0.5, 0.5)	Shocks’ standard errors
**Tax shock**		
$\rho_{\tau_{f}},\rho_{\tau_{f}}^{*}$	(0.86, 0.86)	Smooth coefficients
$\tau_{f,ss},\tau_{f,ss}^{*}$	(0.2, 0.2)	Tax at the steady states
$\mu_{\tau_{f}},\mu_{\tau_{f}}^{*}$	(0.5, 0.5)	Shocks’ means
$\sigma_{\tau_{f}},\sigma_{\tau_{f}}^{*}$	(0.5, 0.5)	Shocks’ standard errors
**Monetary Policy**		
$\rho_{\pi },\rho_{\pi }^{*}$	(1.25, 1.25)	Reaction coefficient of inflation
**Transaction costs *χ*_*h*_, *χ*_*f*_**		
$\chi_{h0},\chi_{h0}^{*}$	(0.03, 0.2)	Coefficients of home assets
$\chi_{h1},\chi_{h1}^{*}$	(2,2)	Coefficients of home assets
$\chi_{f0},\chi_{f0}^{*}$	(0.13, 0.3)	Coefficients of foreign assets
$\chi_{f1},\chi_{f1}^{*}$	(2, 2)	Coefficients of foreign assets
$Fixcost_{f},Fixcost_{f}^{*}$	(10, 10)	Fixed costs

In the households and firms sector, most of the parameters mainly follow Kaplan et al. [6]. In fact, several recent papers on the HANK model take similar calibration values for households’ optimizations. Substitution elasticity on the price *ϵ*_*p*_ and Share of home price *ρ*_*p*_ are set by the model environment. The other parameters in these two sections follow the Kaplan et al. [6].

For the transaction costs, if home households want to invest in the foreign illiquid assets, they need to pay the extra costs *Fixcost*_*f*_ for international transactions. However, paying these extra costs for households investing in home assets is unnecessary. Here this paper assumes that the two countries fixed costs are similar (10, 10).

This paper assumes that two countries launch tariffs shock on imported goods as following process,

$$\begin{array}{c} \begin{array}{cc} \text{ln}\;\tau_{im,t} & =\left(1-\rho_{\tau_{im}}\right)\text{ln}\;\tau_{im,ss}+\rho_{\tau_{im}}\text{ln}\;\tau_{im,t-1}+\epsilon_{\tau_{im}}, \end{array} \end{array}$$ (29)

where $\rho_{\tau_{im}}$ denotes the smooth coefficient on the steady state of tariffs *τ*_*im*,*ss*_. $\in_{\tau_{im}}∼N\left(\mu_{im},\sigma_{im}^{2}\right)$ is an exogenous series of shock. The smooth coefficients ($\rho_{\tau_{im}}$, $\rho_{\tau_{im}}^{*}$) are (0.86, 0.86) [9]. The values of steady states of tariffs $\tau_{im,ss},\tau_{im,ss}^{*}$, shocks’ means $\left(\mu_{\tau_{i}m},\mu_{\tau_{i}m}^{*}\right)$ and shocks’ standard errors $\left(\sigma_{\tau_{i}m},\sigma_{\tau_{i}m}^{*}\right)$ are set by the model environment. This model can adjust density of shocks by means and standard errors.

Similarly, the two countries’ governments levy the tax for the income of investments on foreign illiquid assets. Tax shocks follow

$$\begin{array}{c} \begin{array}{ccc} r_{af} & =r_{ah}^{*}\left(1-\tau_{ft}\right),\;\text{ln}\;\tau_{f,t} & =\left(1-\rho_{\tau_{f}}\right)\text{ln}\;\tau_{f,ss}+\rho_{\tau_{f}}\text{ln}\;\tau_{f,t-1}+\epsilon_{\tau_{f}}, \end{array} \end{array}$$ (30)

*r*_*af*_ is the interest rate after the tax shocks which is the balance that the foreign country’s interest rate $r_{ah}^{*}$ subtracts tax shock $r_{ah}^{*}\tau_{ft}$. Home households’ interest rate *r*_*af*_ of investments for foreign illiquid assets is decided by the interest rate of illiquid assets in the foreign country. $\rho_{\tau_{f}}$ denotes the percentage of investment tax deviating from the steady state, *τ*_*f*,*ss*_ is the steady state of investment tax for foreign assets *a*_*ft*_, and $\in_{\tau_{f}}∼N\left(\mu_{f},\sigma_{f}^{2}\right)$ is an exogenous series of shock. Like the tariffs shock, the smooth coefficients ($\rho_{\tau_{f}}$, $\rho_{\tau_{f}}^{*}$) are (0.86, 0.86) [9]. Other parameters of the tax shock to foreign investments are set by the model environments.

## 4 The mechanism of two countries’ friction on the trade

In the international trade friction, two countries usually suppress the opponent through tariffs on the prices of imported goods and income of investments on foreign illiquid assets. With a larger economic scale and higher technology level, the country has many advantages in the trade friction. For example, the stronger can limit production factors into the low-tech country and care nothing about the imports from the low-tech country because she can easily find the substitutions.

**Fig 3:**
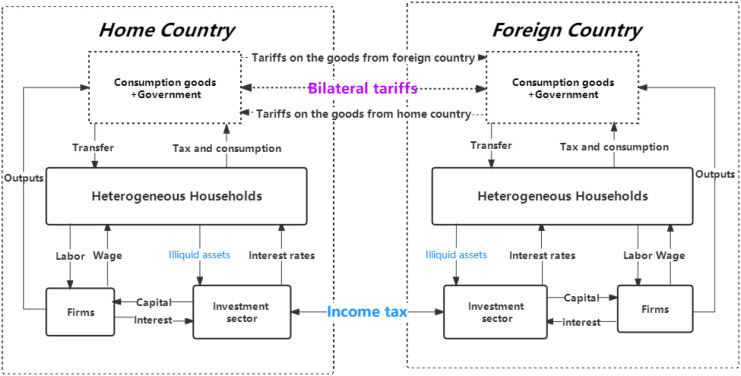
Trade friction mechanism between two countries.

The Fig 3 shows two tariff shocks to consumption goods and investment sectors. First, consumption as the policy function directly decides households’ utilities. Meanwhile, consumption is supported by income and conversely affects the household’s labor supply. If the government levies tariffs on the price of imported goods, households will reallocate their portfolios, such as reducing imported goods or expanding investments on the assets. Second, the home country can levy tariffs on the income from investments in foreign illiquid assets. Then home households achieve less income than before and reduce foreign investments. Subsequently, aggregated illiquid assets held by the foreign country decrease, which may result in foreign outputs falling. Trade friction seems to be only obvious to households’ consumptions and illiquid assets, but it also can be transmitted to other two countries’ aggregated economic variables and impacts on heterogeneous households’ wealth distribution. This paper defines three cases on the two countries’ trade friction.

**Definition 2** *Case1: home country levies tax on the foreign country.*

Regardless of countries’ scale, the home country firstly launches the trade war and levies tax on the foreign country. However, the countries’ scale and economic structure will decide who the winner is.

**Definition 3** *Case2: foreign country levies tax on the home country.*

Similar to Case1, Case2 also means that the foreign country firstly launches the trade war and levies tax on the home country. Which one firstly launches the trade war is indifferent if two countries’ scale and economies are symmetric.

**Definition 4** *Case3: Two countries levy tax with each other.*

Unlike case1 and case2, here, two countries are launching the trade war simultaneously. The winner of this trade war depends on their economic scales and structures. This circumstance will be simulated by the co-shocks of tax to the two countries.

**Definition 5** *Symmetric and Asymmetric framework*

**Symmetric framework** means that the home country and foreign country have the same technology level and Capital capacity(Liquid assets and Illiquid assets); **Asymmetric framework** means that the home country has a lower technology level than the foreign country, the foreign country has a larger capital capacity than the home country.

## 5 Symmetric economy

**Fig 4:**
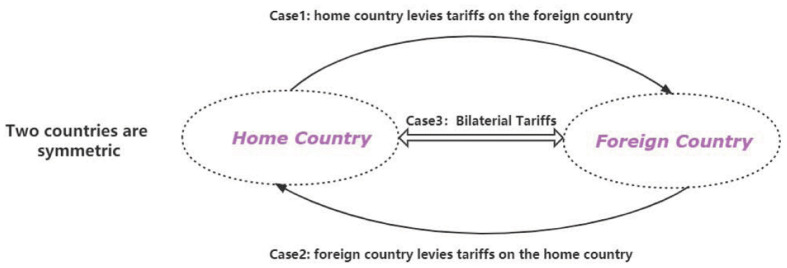
Trade war under the symmetric economy.

As the definition and Fig 4, we discuss three cases under the environment where two countries are symmetric. The two countries have similar technology levels, economic scales, and structures. For example, the two countries’ aggregated illiquid and liquid assets are close. They also have similar distributions on their steady states. In the simulation process, we use the same parameters on these two countries. Meanwhile, in this symmetric environment, two countries have the same market mechanisms. This section mainly simulates the three cases above and gives corresponding impulse responses figures.

### 5.1 Two countries’ policy analysis

We characterize the impacts of the trade war in the HANK model rather than the homogeneous model because the HANK relaxes assumptions on the representative agents. Thus we can explore the heterogeneous households’ reactions and economic behaviors under the environments of rising tariffs. Tariffs are the primary channel to start a trade war.

**Fig 5:**
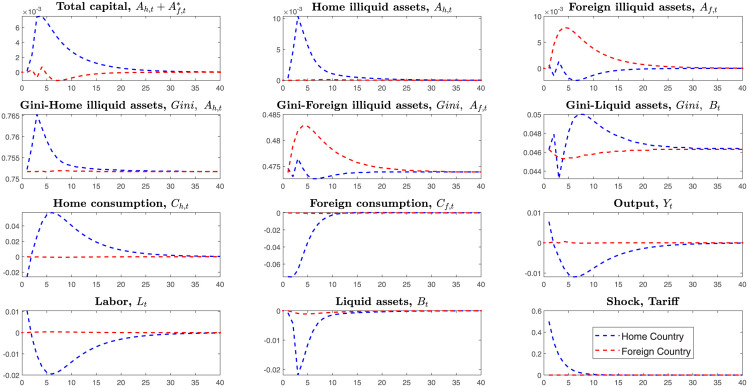
Impulse responses, under the tariffs shock of home country.

Hence, we simulate two countries’ economies through the tariffs shock. The Fig 5 simulates the *case1*, home country firstly levies tariffs on the imported goods from the foreign country in the environment that two countries are symmetric.

This section assumes that the home country firstly launches the trade war, raises tariffs for the imported goods, and the foreign country passively reacts to the home country’s tariffs shock.

The home country’s economic variables act as follows on the transmitting channels of tariffs shock in Fig 5. Tariffs first enter into household consumption(policy function) and result in rising prices of imported goods, and households will consume less imported goods and then purchase more home goods. In this model, households will allocate income over liquid assets, consumption, and illiquid assets. Therefore the dominant liquid assets also reduce subsequently. As the home consumption demand expands, the total supply(outputs) also will be stimulated. Households invest more in the home illiquid assets and less in the foreign illiquid assets. The home total illiquid assets $A_{h}+A_{f}^{*}$ still increase, but the home households supply fewer labors, and the total outputs decrease sharply.

On the losses on two countries, in the *Case1*, we measure the loss on two countries’ total illiquid assets, liquid assets, consumption, and outputs. After raising tariffs on the imported goods, the home country also reduces investments on the foreign illiquid assets *A*_*f*_ subsequently. Finally, the home outputs reduce more than the foreign country. The foreign country’s economic variables fluctuate less than the home country when the home country raises tariffs. Home country’s higher imported tariffs mainly affect foreign country’s export if the foreign country does not raise imported tariffs. Compared to the home country’s decreased outputs, foreign country’s economic variables only reduce a little because the decreasing export does not largely affect the foreign country’s home industry, especially for the country with enough large home market.

The economic system is complicated, not simply decided by the tariffs only. In the transmitting process of tariffs, differences between two countries’ economic structures also affect these results. However, here we assume that two countries have similar scales and structures. Thus, in the symmetric framework, the home country firstly launched tariffs on imported goods suffered larger losses than the foreign country.

Like the *Case1*, here we simulate the *Case2*, foreign country launches the trade war and levies the unilateral tariffs on the imported goods from home country.

**Fig 6:**
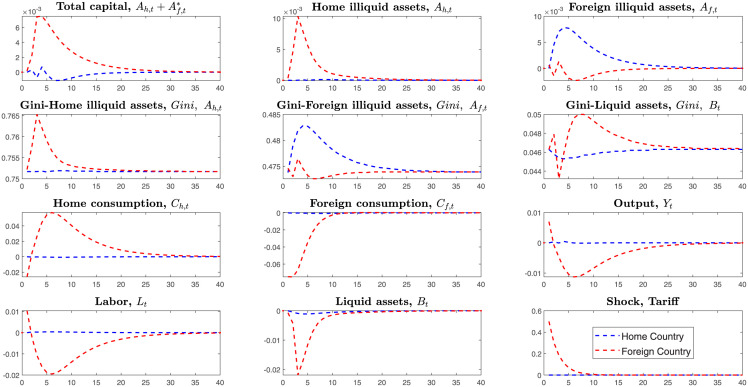
Impulse responses, under the tariffs shock of foreign country.

In the symmetric economic system, the foreign country gets the same results as the home country if he first launches the trade war and raises the tariffs. Thus, from the impulse responses in Fig 6, we know that the country that first launches the trade war will suffer more enormous losses when two countries have symmetric economic structures.

In the dynamic games, the opponent may strike back the first tariffs launcher and raise tariffs on imported goods. Thus we next simulate the *Case3*, two countries simultaneously levy tariffs with each other.

**Fig 7:**
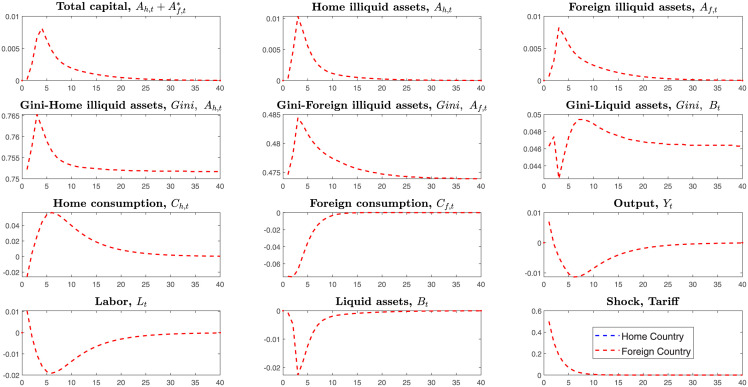
Impulse responses, under the bilateral tariffs shocks.

What will happen if two countries both launch the bilateral tariffs shocks for their opponents? Unlike the *Case1* and *Case2*, here we simulate the *Case3* that two countries simultaneously raise tariffs for the imported goods. From the Fig 7, we can find two countries’ dominant economic variables fluctuates negatively. Under the bilateral tariffs shocks, all the variables in the model transmit as follows. Two countries’ households initially hold large amounts of disposable income because their consumption reduces. As consumption for foreign goods decreases, home demand is stimulated. Then households invest more in the home illiquid assets and foreign illiquid assets. Subsequently, the stock of liquid assets also decreases.

Launching bilateral tariffs will make both countries suffer more losses than unilateral tariffs in the symmetric economic system. They cannot get higher benefits from levying tariffs in the symmetric system because two countries rely on each other. That is, it is unintelligent to launch a trade war if two countries have similar economic scale and structure.

Notably, unlike the Tow-Country RA framework, the Two-Country HA model depicts changes of Gini-coefficients on the heterogeneous households’ investments on the liquid and illiquid assets. The figures above on the Gini-coefficients indicate that after bilateral tariffs shock in the symmetric framework, households’ inequalities deteriorated more than before. This trade war will make the two countries’ households’ wealth distribution imbalanced. They have no motivation to launch the trade war under the symmetric system in the long run.

## 6 Asymmetrical economy

**Fig 8:**
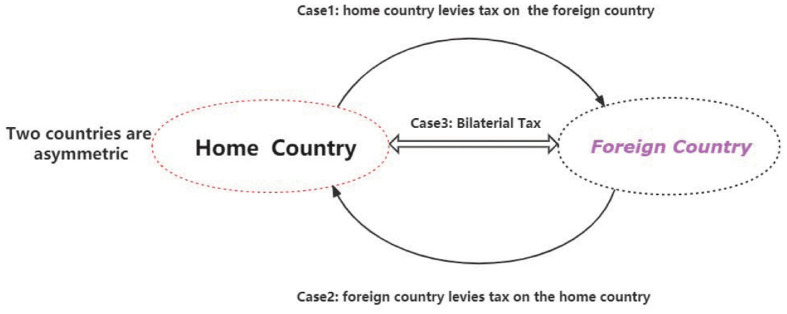
Trade war under the asymmetric economy.

As shown above in the Fig 8, we simulate the two countries under an asymmetric environment where the two countries’ technology productivities, economic scales, and structures are different.

Usually, two countries are asymmetric and have distinct gains and losses from the trade war. For the Asymmetrical Economy, we assume that there are two countries. The home country is low-tech, and the foreign country has higher technology. Foreign country’s households have higher productivities and larger capital stock of illiquid assets than the home country. In contrast, home country’s households have lower productivities and smaller capital stock of illiquid assets. Two countries trade in an asymmetrical system, and the foreign country has more relative advantages than the home country.

In the asymmetric framework, we will simulate two cases. *Case2*, what will happen if the foreign country(high-tech) firstly launches the tariffs shock? *Case3*, what will happen if these two countries both simultaneously launch the tariffs shocks?

Finally, we will do an exceed bilateral tax shock to the income of investments on illiquid assets and imported goods. Unlike the shocks of tariffs to the imported goods, these shocks simulate economies from the consumption sides and production sides.

To explore the impacts of tariffs shock of the foreign country(high-tech country) on the home country(low-tech country), we simulate the tariffs shock in the *Case2*.

**Fig 9:**
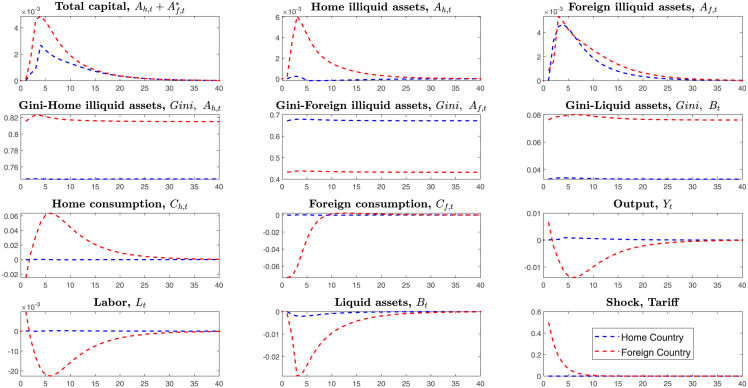
Impulse responses, under the tariffs shock of foreign country.

On the transmitting channels of tariffs shock, like the symmetric system, tariffs firstly transmit from the household’s consumption to the amounts of liquid and illiquid assets, then to their labor supply, and finally affect the firm’s production. As Fig 9 shows, the foreign country levies tariffs on the imported goods from the home country, and then domestic consumption and investments on illiquid assets both rise, while consumption for imported goods decreases. Households reallocate their liquid assets at investments and consumption and supply less labor, resulting in decreasing outputs. Like the symmetric framework, regardless of the low or high-tech country, it is difficult to win the trade war only using the tariffs tool on the imported goods. Besides, the liquid and illiquid assets inequality have become larger than before.

Compared to the symmetric framework, the foreign country, as a high-tech country that firstly launches tariffs shock, suffers more outputs losses than the low-tech country only by unilateral levying tariffs on the imported goods.

For the low-tech country, technological progress is an important means to relieve the recession caused by trade frictions. Rising technology may stimulate investments in illiquid assets and expand the consumption of home goods. On the economic supply side, the aggregated outputs are decided by technology, investments, and labor. Therefore, raising technology first attracts investments in the illiquid assets of the home country. Home firms will produce more high-tech products, and then households can improve their utilities by expanding consumption of these goods. As the stock of capital in the short term is finite, home households will reduce foreign investments in illiquid assets. Meanwhile, foreign households also increase investments in the home illiquid assets since they can achieve higher capital returns from investments in high-tech enterprises. Hence, this technological progress can make the low-tech country change its dilemma and boost the economic recovery on the aggregate demand side.

**Fig 10:**
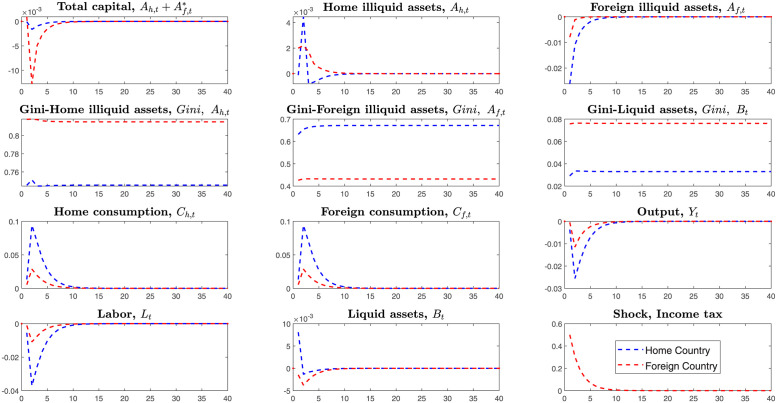
Bilateral shocks of income tax on foreign investments.

To explore the impacts of bilateral tax between the home country and foreign country in the asymmetric framework, we simulate the *Case3* that home country and foreign country simultaneously levy tax on households income from investments on foreign illiquid assets in the Fig 10.

In this *Case3*, the home country levies the tax on households’ income from investments for the foreign illiquid assets. Then home households will invest less in the foreign illiquid assets, which means that households have fewer income channels and reduce foreign investments.

In the home country(low-tech), households’ income mainly consists of several sections: income of investments for liquid assets, home and foreign illiquid assets, and labor supply. All the capitals that initially invested on the foreign illiquid assets will return to the home and reallocate at the consumption and home illiquid assets. Subsequently, the home labor supply reduces, and outputs also decrease. In the asymmetric system, like the home country, the foreign country(high-tech) also levies the tax on the income of investments on foreign illiquid assets. Similarly, the capital also will return to the home and stimulate consumption. However, the degree of capital returning in the foreign country is smaller than in the home country.

On degrees of losses for two countries, two sides both suffer losses. However, the home country suffers losses higher than that of the foreign country. In this model, outputs are decided by productivity, total capital, and labor supply. The home country has lower productivity and economic scale and invests overly in foreign illiquid assets. Home households will retract investments on the foreign illiquid assets and seem to be richer than before after the home government levies income tax on investments on the foreign illiquid assets. Thus home households reduce labor supply more than that of foreign households.

Therefore, both countries will suffer losses in the trade war from levying income tax on investments on the foreign illiquid assets, but the foreign country will suffer smaller losses.

**Fig 11:**
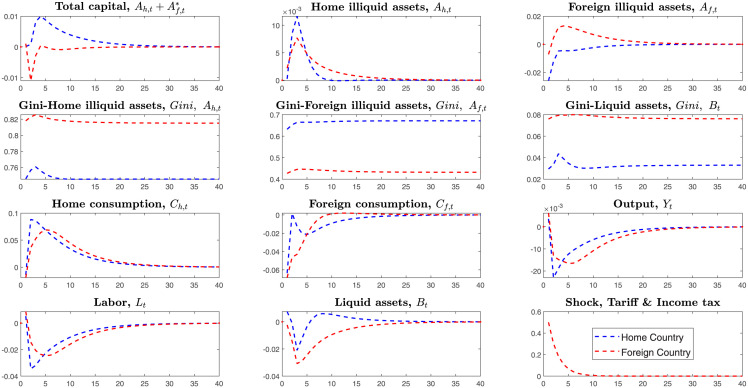
Bilateral shocks on the tariffs and income tax of foreign investments.

Unlike all the shocks of before, this section simulates the bilateral co-shocks to the mutual income of investments and mutual imported goods in the Fig 11.

Households in the home country(Low-tech) invest more in illiquid assets of the foreign country than those households in the foreign country(High-tech) invest in the illiquid assets of the home country $A_{f}>A_{f}^{*}$.

Home country’s total illiquid assets consist of home illiquid assets and foreign investments on the home illiquid assets, $A_{h}+A_{f}^{*}$. Similarly, the foreign country’s total illiquid assets also consist of home illiquid assets and home investments on the foreign illiquid assets, $A_{h}^{*}+A_{f}$. In international trade, levying tax on the income of investments on illiquid assets is to cut return rates of illiquid assets.

Here, the tax on the income of investments and imported goods transmit as follows. Firstly, confronting the bilateral tariffs on the mutual imported goods, two countries’ households both reduce consumption for the imported goods and expand home consumption. In the short turn, decreasing consumption of imported goods makes households liquid assets return to the home and purchase more domestic goods. Meanwhile, households will supply less labor because of the sudden decrease in imported goods. Secondly, if two countries’ governments levy income tax on foreign investments, the low-tech country will retract more foreign investments and expand home investments. From Fig 11, we know that the home country(low-tech country) largely retract investments on foreign illiquid assets, expand consumption and finally reduce more labor supply than that of the foreign country.

The home country’s economy fluctuates higher than the foreign country. The foreign country suffers lower losses on outputs. However, the high-tech country’s Gini-coefficients become higher than before. That is, in the trade war, if two countries levy tariffs on the imported goods and income tax on foreign investments simultaneously, the high-tech country will suffer lower loss, but the inequality on the illiquid and liquid assets will become higher than before.

## 7 Analysis of trade data on US and China

For the mechanism of trade war analyzed in sections 5 and 6, a similar example in the asymmetric framework is trade friction between China and US. Here we collect data on the changes in trade after and before the trade war and reveal the economic logic. The data contains the changes in imports, GDP growth, and import subdivision over five years.

**Fig 12:**
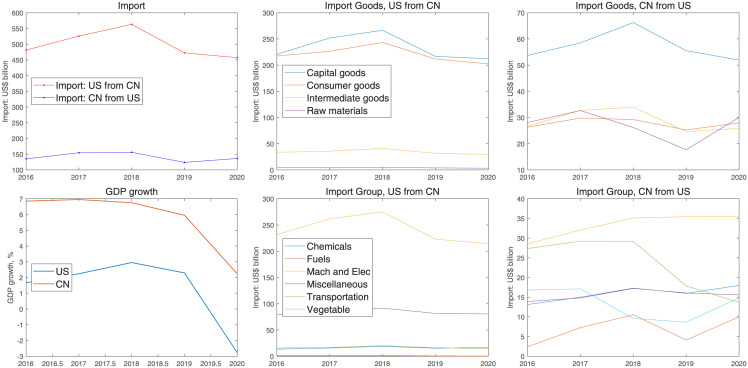
Changes on imported goods before and after trade friction.

Fig 12 shows that the two countries’ imports decreased after the trade war. The decreasing degree of US imports is relatively higher than that of China. In terms of the outputs, before 2019, the growth rate of GDP in the US was slower than that in China. This downward trend is caused by two factors. On the one hand, rising tariffs reduce the opponent’s exports and then transmit to the produce side; On the other, COVID-19 limited consumption and investment growth in the economy. The import group contains Chemicals, Fuels, Mach and electronics, Miscellaneous, Transportation, and Vegetable. The Mach and Electronic goods that the US imported from China are largely more than that of China since the US limited the export of high-tech goods to China. Except for the common consumption goods, the core of trade friction between the US and China is the exported high-tech goods. In the macro-system, the outputs are decided by technology, investments, and labor supply. Investments and technology are separately affected by capital goods and high-tech products. Capital goods are an important part of illiquid assets, which are mainly affected by the return rates and investment taxes. In the asymmetric trade framework, the US obviously has a higher level of high technology, and China’s high-tech industrial development is dependent on American semiconductor technology. Therefore, once the United States restricts the export of high-tech products, it will affect China’s industrial transformation and upgrading. Meanwhile, if China raises tariffs on other common imported goods from the US, the power of such countermeasures is not obvious since other countries may import other common goods from the US.

Fig 12 reveals the essence of the Sino-US trade from two aspects. The first is the trade imbalance between China and the United States. The import and export trade between China and the United States presents a huge trade deficit, and China’s exports to the US far exceed its imports to the US. Before or after trade frictions, the total imports of the United States from China were greater than China’s imports from the United States. The second is that China and the United States have unbalanced positions in the global industrial chain and competition in the high-tech product market. Before the year 2000, the United States was responsible for the research and development and production of high-tech products at the core, while China was mainly responsible for the production of ordinary daily necessities and middle and low-end products. China’s exports to the United States have a higher proportion of capital and labor-intensive products. In fact, China, in the production of mechanical and electrical equipment, did not master the core technology of these products. However, in the last decade, China has begun to produce high-tech products and has seized the market for high-tech products that originally belonged to the United States. The US high-tech export ban has cut off exports of the most skilled high-tech means of production to China. In the asymmetric framework, the high-tech country exports high-tech goods, and the low-tech country exports low-end products, and then the two sides reach a steady state in the division of labor in the industrial chain. The low-tech countries imported high-tech means of production, such as chips, and produced high-tech products to seize the market of high-tech countries, breaking this steady state. These two factors above are the root cause of the outbreak of Sino-us trade friction.

Overall, the actual data on the economic fluctuations after trade friction are basically consistent with the model simulation results. Limiting high-tech goods exported to China has reduced China’s output. In trade frictions, the United States, with higher technological levels, suffers fewer losses, while China’s trade situation is more passive.

## 8 Third-party countries

This section considers this case: What will happen if a country in a trade war transfers its trade to a third party? In March 2019, the United States raised its average tariff rate on China from 0.1 to 0.25; In May of the same year, China also raised its average tariff rate on the United States to 0.25. The escalation of trade frictions has caused the residents of both countries to bear most of the tariff increases, which will lead to the transfer of some trade to third countries. Due to geographical location, production costs, and trade policies, this trade transfer effect is most likely to occur in Southeast Asian countries.

**Fig 13:**
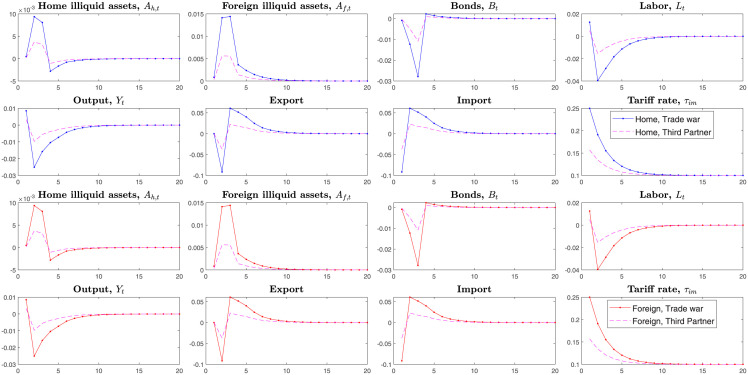
Comparison between the trade war and third-party trade detours.

Trade detour means that some foreign trade will bypass the country launching tariff shock to avoid excessive direct tariffs. Meanwhile, This trade detour can avoid higher tariffs, but it will also bring corresponding costs, such as transportation costs and transaction costs. In order to describe the difference between the direct trade war and the trade detour with the third country, we simulate these two cases in the model. The simulating results are shown in the following figure:

Fig 13 shows the simulating results of two cases: The first is that China and the US levies tariffs on each other; The second is that both countries’ trade detours through Vietnam. When there are no trade detours, the two countries impose each other from 10 percent tariff on imported goods to 25 percent; however, when there is a third-partner country, such as Vietnam, Vietnam levies about 5.68 percent tariffs on imported goods from China, and the US. Thus, as long as the cost of trade detours to Vietnam is less than 25 percent, the trade detour is profitable. From the IRF figure, the direct trade war would reduce China’s total output by 2.5 percent and make US’s output decrease by 1.7 percent. However, the trade detour makes the output of China and the US separately decrease by 0.8 and 0.5 percent. Once a trade detour occurs, it will reduce the loss suffered from the trade war.

These simulated results show a comparison between two countries in the trade war and trade detours with the third country. Trade detours substantially reduce tariff levels and have a significantly lower impact on total output than in the case of direct trade wars, thereby reducing losses. However, trade detours only occur when the sum of the costs of trade detours and tariffs is less than the tariffs from the trade war.

A typical example of a trade detour is the Vietnamese Photovoltaic industry: as the US levies tariffs on China’s Photovoltaic products, some Chinese manufacturers have therefore moved their product assembly lines to Vietnam, which has brought some of China’s production capacity back to the European and American markets in the image of Vietnamese exports. This is an example of a trade detour that led to industrial transfer and proved that pure import and export restrictions could not completely kill a country’s industry.

We introduce the actual data from US, China, and Vietnam as an example to show the Trade Diversion Effect. These data mainly consist of aggregation and subdivision of imported goods among three countries.

**Fig 14:**
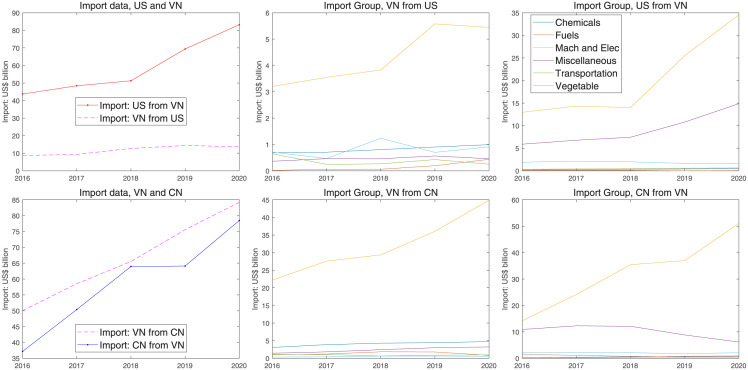
Trade data on the three countries.

This section considers the third partner’s role in international trade. Fig 14 shows that the US increased the imported goods from Vietnam, especially machinery and electronic products, after the trade friction. The actual data and model simulation results show that the US achieved higher returns through the trade Detour.

As can be seen in the figure, after 2018, the number of mechanical and electronic products imported by China and the United States from Vietnam increased rapidly, but the total amount of mechanical and electronic products imported by Vietnam from the United States was very low, while the total amount of mechanical and electronic products imported from China was very high and increased rapidly. We can obtain two guesses from the above data: 1. The total amount of machinery and electronic products imported from China and the US is asymmetric. Before the trade friction between China and the United States, Vietnam’s domestic market demand for mechanical and electronic products was low; after the trade friction, the demand in Vietnam increased. Some mechanical and electronic products produced in China entered the US market through Vietnam; 2, some of China’s machinery and electronics production capacity was transferred to Vietnam during the trade war. After the year 2019, the total amount of mechanical and electronic products imported from China to Vietnam increased rapidly, and the US imported goods from Vietnam also quickly raised. It is most likely that Chinese mechanical and electronic products have entered the US market through Vietnam.

## 9 Conclusions

This paper makes several marginal contributions separately from the technical, theoretical, and empirical perspectives. First, this paper provides a heterogeneous framework for analyzing trade friction between countries with symmetric and asymmetrical economic structures. This heterogeneous assumption is a big step for describing the wealth distribution, compared with the homogeneous assumption in the Galí and Monacelli [2]. This progress, in fact, expands the research boundary of the New Keynesian model and may improve the accuracy of forecasts of international macroeconomic policy; Second, this paper’s two-country framework can depict the changing mechanism of the bilateral interest rate and investment and is an advantage, compared with the SOE-HANK model in the Auclert et al. [1]. This advantage will provide the technical support and theoretical basis for the study of the endogenous interaction in the international financial market; Third, the Two-country HANK framework provides a reference paradigm for analyzing international multilateral trade frictions. This model can evaluate the tariffs policy from multiple perspectives, including two countries’ consumption, investments, productivity, labor supply, and wealth inequality, rather than only analyzing the domestic economic changes in Oskolkov [20]; Fourth, the condition and conclusion on the trade detour will provide a new thought-way when analyzing the bilateral trade friction and expanding the Two-country HANK model.

Based on the research results, this paper makes the following policy suggestions for WTO(World Trade Organization) in establishing a more efficient and perfect multilateral trading system to stimulate the recovery and growth of the global economy ultimately: 1. Avoid the low-tech countries from falling into technical dilemmas by promoting developed and developing countries to reach industrial transfer agreements. It can be seen from the HANK model that the stimulus effect of technology shock is higher in low-tech countries than that in high-tech countries. Therefore, transferring high-tech industries to low-tech countries can enlarge the scale of middle-class consumer markets, thus expanding global demand; 2. Strengthen the coordination and settlement of trade disputes and shorten the cycle of trade frictions. The simulation results of this paper show that the economic losses caused by the trade war and the duration of the trade war are positively correlated. The coordinating time of WTO should be shorter in the trade war between China and the United States. A long coordination cycle will reduce the cost for the large countries to use trade wars to suppress the small countries and expand the negative impact of the trade war on the global economy; 3. Promote balanced trade and balanced distribution of industrial chains. It is found that a large trade deficit is an important motivation for the launch of trade wars, and the unbalanced industrial distribution is also the premise for high-tech countries to set technical barriers to low-tech countries. Therefore, making different countries interdependent on the industrial chain is one of the effective measures to avoid trade wars.

At present, the two-country HANK model is mainly used in the field of theoretical analysis. However, the futuristic model integrated into the Bayesian estimation of actual data will be an important direction and have more powerful explanatory abilities.

## 10 Appendix

### 10.1 Algorithm to solve Two-Country HANK model

This section introduces the algorithm to solve the Two-Country HANK model. Technically, for the two countries’ framework, our main contributions lie in developing the HJB equations with three heterogeneous assets and solving it, based on Kaplan et al. [6].

In the HANK model, the results of optimization on the firm sector are the wage and return rates of illiquid assets which can be taken into households balance sheets. Thus, optimizations of two sectors, households and firms, are factually finished by the optimization for households.

Time is continuous. There are continuous households in the economy, holding liquid assets *b*_*t*_, domestic illiquid assets *a*_*ht*_, foreign illiquid assets *a*_*ft*_, and personal productivity *z*_*t*_. The households’ optimization problem is given by:

$$\begin{array}{c} \begin{array}{cc} & \;\underset{c_{ht},c_{ft},\ell_{t}}{\text{max}\;}\int_{t=0}^{\infty }u\left(c_{ht},c_{ft},l_{t}\right)dt,\;u=\frac{{\left(c_{ht}\right)}^{1-\gamma_{c}}}{1-\gamma_{c}}+\varphi_{c}\frac{{\left(c_{ft}\right)}^{1-\gamma_{c}}}{1-\gamma_{c}}-\varphi_{\ell }\frac{{\left(\ell_{t}\right)}^{1+\gamma_{\ell }}}{1+\gamma_{\ell }}\\ s.t. & \\ & \dot{b_{t}}=\left(1-\tau_{w}\right)w_{t}\ell_{t}z_{t}+r_{b}b_{t}+T_{t}-d_{ht}-\chi_{h}\left(d_{ht},a_{ht}\right)-d_{ft}-\chi_{f}\left(d_{ft},a_{ft}\right)-c_{ht}\frac{P_{ht}}{P_{t}}\\ & -c_{ft}\frac{P_{ft}e_{t}\left(1+\tau_{im}\right)}{P_{t}},\\ & \dot{a_{ht}}=r_{ah}a_{ht}+d_{ht},\\ & \dot{a_{ft}}=r_{af}a_{ft}+d_{ft}\frac{P_{t}}{P_{t}^{*}e_{t}},\\ & dz_{t}=-g_{j}z_{t}dt+\epsilon_{t}dW_{t},\;\epsilon_{t}∼N(0,\sigma_{z}^{2}).\\ & \chi_{h}\left(d_{ht},a_{ht}\right)=\chi_{h0}\left|d_{ht}\right|+\chi_{h1}{\left|\frac{d_{ht}}{\text{max}\{a_{ht},\underline{a_{h}}\}}\right|}^{2}\text{max}\;\left\{a_{ht},\underline{a_{h}}\right\}\\ & \chi_{f}\left(d_{ft},a_{ft}\right)=Fixcost_{f}+\chi_{f0}|d_{ft}\frac{P_{t}}{P_{t}^{*}e_{t}}|+\chi_{f1}{\left|\frac{d_{ft}\frac{P_{t}}{P_{t}^{*}e_{t}}}{\text{max}\{a_{ft},\underline{a_{f}}\}}\right|}^{2}\text{max}\;\left\{a_{ft},\underline{a_{f}}\right\} \end{array} \end{array}$$ (31)

#### 10.1.1 Endogenous consumption for imported goods

In this subsection, we introduce how to simplify the utility function and assume that there is a total consumption *c*_*t*_ of the heterogeneous households, satisfying:

$$\begin{array}{c} c_{t}P_{t}=P_{ht}c_{ht}+P_{ft}e_{t}\left(1+\tau_{im}\right)c_{ft} \end{array}$$ (32)

The total price level *P*_*t*_ in the home country is composed of domestic goods price *P*_*ht*_ and price of imported goods *P*_*ft*_*e*_*t*_(1 + *τ*_*im*_):

$$\begin{array}{c} P_{t}={\left[\rho_{p}{\left(P_{ht}\right)}^{\varepsilon_{p}}+\left(1-\rho_{p}\right){\left(P_{ft}e_{t}\left(1+\tau_{im}\right)\right)}^{\varepsilon_{p}}\right]}^{\frac{1}{\varepsilon_{p}}}, \end{array}$$ (33)

where *ρ*_*p*_ denotes the share of domestic goods price, and *ε*_*p*_ is the elasticity between domestic and foreign goods price.

For households’ optimization problems, the Hamilton-Jacobi-Bellman equation is:

$$\begin{array}{c} V\left(b_{t},a_{ht},a_{ft},z_{t}\right)=\underset{c_{ht},c_{ft},\ell_{t}}{\text{max}\;}u\left(c_{ht},c_{ft},l_{t}\right)+V_{b}\dot{b_{t}}+V_{ah}\dot{a_{ht}}+V_{af}\dot{a_{ft}}+g_{z}V_{z}+\frac{\sigma_{z}^{2}}{2}V_{zz} \end{array}$$ (34)

where *V*(*b*_*t*_, *a*_*ht*_, *a*_*ft*_, *z*_*t*_) is the value function of households, *V*_*b*_, *V*_*ah*_, *V*_*af*_, *V*_*z*_, *V*_*zz*_ are the partial derivatives of the value function.

The partial derivatives of the HJB equation over *c*_*ht*_, *c*_*ft*_ are

$$\begin{array}{c} \begin{array}{cc} & 0=c_{ht}^{-\gamma_{c}}-\frac{P_{ht}}{P_{t}}V_{b}\\ & 0=\varphi_{c}c_{ft}^{-\gamma_{c}}-\frac{P_{ft}e_{t}\left(1+\tau_{im}\right)}{P_{t}}V_{b} \end{array} \end{array}$$ (35)

which means that *c*_*ht*_ and *c*_*ft*_ are connected by their relative prices

$$\begin{array}{c} c_{ft}=c_{ht}{\left[\frac{P_{ft}e_{t}\left(1+\tau_{im}\right)}{\varphi_{c}P_{ht}}\right]}^{-\frac{1}{\gamma_{c}}} \end{array}$$ (36)

Substitute this equation above into the total consumption *c*_*t*_, then we have:

$$\begin{array}{c} \begin{array}{cc} & c_{ht}=\frac{c_{t}P_{t}}{P_{ht}+{\left[P_{ft}e_{t}\left(1+\tau_{im}\right)\right]}^{1-\frac{1}{\gamma_{c}}}{\left(\varphi_{c}P_{ht}\right)}^{\frac{1}{\gamma_{c}}}}\\ & c_{ft}=\frac{c_{t}P_{t}}{{\left[P_{ft}e_{t}\left(1+\tau_{im}\right)\right]}^{\frac{1}{\gamma_{c}}}{\left(\varphi_{c}P_{ht}\right)}^{-\frac{1}{\gamma_{c}}}P_{ht}+P_{ft}e_{t}\left(1+\tau_{im}\right)} \end{array} \end{array}$$ (37)

Substitute these equations *c*_*ht*_ and *c*_*ft*_ above into the utility function before and have

$$\begin{array}{c} \begin{array}{cc} & \;\;\;\;\;\;u(c_{ht},c_{ft},\ell_{t})\\ & =\frac{{\left(c_{ht}\right)}^{1-\gamma_{c}}}{1-\gamma_{c}}+\varphi_{c}\frac{{\left(c_{ft}\right)}^{1-\gamma_{c}}}{1-\gamma_{c}}-\varphi_{\ell }\frac{{\left(\ell_{t}\right)}^{1+\gamma_{\ell }}}{1+\gamma_{\ell }}\\ & =\frac{{\left(c_{t}\right)}^{1-\gamma_{c}}}{1-\gamma_{c}}{\left[\frac{P_{t}}{P_{ht}+P_{ft}e_{t}\left(1+\tau_{im}\right){\left[\frac{P_{ft}e_{t}\left(1+\tau_{im}\right)}{\varphi_{c}P_{ht}}\right]}^{-\frac{1}{\gamma_{c}}}}\right]}^{1-\gamma_{c}}(1+\varphi_{c}{\left(price_{ch,cf}\right)}^{1-\gamma_{c}})\\ & -\varphi_{\ell }\frac{{\left(\ell_{t}\right)}^{1+\gamma_{\ell }}}{1+\gamma_{\ell }}\\ & =\alpha_{c}\frac{{\left(c_{t}\right)}^{1-\gamma_{c}}}{1-\gamma_{c}}-\varphi_{\ell }\frac{{\left(\ell_{t}\right)}^{1+\gamma_{\ell }}}{1+\gamma_{\ell }},\;\text{where}\;\\ \alpha_{c} & \equiv {\left[\frac{P_{t}}{P_{ht}+P_{ft}e_{t}\left(1+\tau_{im}\right){\left[\frac{P_{ft}e_{t}\left(1+\tau_{im}\right)}{\varphi_{c}P_{ht}}\right]}^{-\frac{1}{\gamma_{c}}}}\right]}^{1-\gamma_{c}}(1+\varphi_{c}{\left[\frac{P_{ft}e_{t}\left(1+\tau_{im}\right)}{\varphi_{c}P_{ht}}\right]}^{\frac{\gamma_{c}-1}{\gamma_{c}}}) \end{array} \end{array}$$ (38)

#### 10.1.2 Endogenous labor supply

According to Benjaming Moll’s website, this section introduces how to derive the endogenous labor supply equation (https://benjaminmoll.com/codes/). Beginning with the HJB equation with the simplified utility function:

$$\begin{array}{c} \begin{array}{cc} V(b_{t},a_{ht},a_{ft},z_{t})=\; & \underset{c_{t},\ell_{t}}{\text{max}\;}u\left(c_{t},l_{t}\right)+V_{b}\dot{b_{t}}+V_{ah}\dot{a_{ht}}+V_{af}\dot{a_{ft}}+g_{z}V_{z}+\frac{\sigma_{z}^{2}}{2}V_{zz}\\ s.t.\;\dot{b_{t}}=\; & \left(1-\tau_{w}\right)w_{t}\ell_{t}z_{t}+r_{b}b_{t}+T_{t}-d_{ht}-\chi_{h}\left(d_{ht},a_{ht}\right)\\ & -d_{ft}-\chi_{f}\left(d_{ft},a_{ft}\right)-c_{t},\\ u(c_{t},l_{t})=\; & \alpha_{c}\frac{{\left(c_{t}\right)}^{1-\gamma_{c}}}{1-\gamma_{c}}-\varphi_{\ell }\frac{{\left(\ell_{t}\right)}^{1+\gamma_{\ell }}}{1+\gamma_{\ell }}. \end{array} \end{array}$$ (39)

Where *V*_*b*_, *V*_*ah*_, *V*_*af*_, *V*_*z*_ are first-order partial derivatives of the value function *V*, and *V*_*zz*_ is the second-order partial derivative. The HJB equations’ first order conditions are given by:

$$\begin{array}{c} \begin{array}{cc} 0 & =\alpha_{c}c_{t}^{-\gamma_{c}}-V_{b}\\ 0 & =-\varphi_{\ell }\ell_{t}^{\frac{1}{\varphi_{\ell }}}+V_{b}\left(1-\tau_{w}\right)w_{t}z_{t} \end{array} \end{array}$$ (40)

Obviously, the labor supply *ℓ*_*t*_ is the function of total consumption *c*_*t*_:

$$\begin{array}{c} \ell_{t}={\left(c_{t}\right)}^{-\gamma_{c}\varphi_{\ell }}{\left[\frac{\alpha_{c}\left(1-\tau_{w}\right)w_{t}z_{t}}{\varphi_{\ell }}\right]}^{\varphi_{\ell }} \end{array}$$ (41)

Consider the budget constraint equation $\dot{b}$. In the equilibrium, changing rates of all assets are equal to zero, and the deposits are also equal to zero. Thus the consumption *c*_0_ and labor supply *ℓ*_0_ is given by:

$$\begin{array}{c} c_{0}=\left(1-\tau_{w}\right)w_{t}\ell_{0}z_{t}+r_{b}b_{t}+T_{t}-fixedcost_{f} \end{array}$$ (42)

Substitute *c*_0_ into the previous equation, and get

$$\begin{array}{c} \ell_{0}={\left(\left(1-\tau_{w}\right)w_{t}\ell_{0}z_{t}+r_{b}b_{t}+T_{t}-fixedcost_{f}\right)}^{-\gamma_{c}\varphi_{\ell }}{\left[\frac{\alpha_{c}\left(1-\tau_{w}\right)w_{t}z_{t}}{\varphi_{\ell }}\right]}^{\varphi_{\ell }} \end{array}$$ (43)

This equation above explains how the labor supply *ℓ*_0_ is determined in the equilibrium.

#### 10.1.3 Algorithm for HJB

Let’s return to the newest version of HJB equation:

$$\begin{array}{c} \begin{array}{cc} V(b_{t},a_{ht},a_{ft},z_{t})=\; & \underset{c_{t},\ell_{t}}{\text{max}\;}u\left(c_{t},l_{t}\right)+V_{b}\dot{b_{t}}+V_{ah}\dot{a_{ht}}+V_{af}\dot{a_{ft}}+g_{z}V_{z}+\frac{\sigma_{z}^{2}}{2}V_{zz}\\ s.t.\;\dot{b_{t}}=\; & \left(1-\tau_{w}\right)w_{t}\ell_{t}z_{t}+r_{b}b_{t}+T_{t}-d_{ht}-\chi_{h}\left(d_{ht},a_{ht}\right)\\ & -d_{ft}-\chi_{f}\left(d_{ft},a_{ft}\right)-c_{t},\\ u(c_{t},l_{t})=\; & \alpha_{c}\frac{{\left(c_{t}\right)}^{1-\gamma_{c}}}{1-\gamma_{c}}-\varphi_{\ell }\frac{{\left(\ell_{t}\right)}^{1+\gamma_{\ell }}}{1+\gamma_{\ell }}. \end{array} \end{array}$$ (44)

According to Achdou et al. [21], the income flow of liquid assets ${\dot{b}}_{t}$ can be split into three items:

$$\begin{array}{c} \begin{array}{cc} & S^{c}=\left(1-\tau_{w}\right)w_{t}l_{t}z_{t}+r_{b}b_{t}+T_{t}-c_{t}\\ & S^{dh}=-d_{ht}-\chi_{h}\left(d_{ht},a_{ht}\right),\;S^{df}=-d_{ft}-\chi_{f}\left(d_{ft},a_{ft}\right)\\ & \dot{b_{t}}=S^{c}+S^{dh}+S^{df} \end{array} \end{array}$$ (45)

Let $x^{F+}≜max\left(x,0\right)$ and $x^{B-}≜min\left(x,0\right)$. Then we define *x* = *x*^*F*+^ + *x*^*B*−^. We can also separate constraint conditions into different parts. Let *b*_*t*_, *a*_*ht*_, *a*_*ft*_, *z*_*t*_ be in different grids, and then we could mark a household as (*i*, *j*, *k*, *nz*) when *b*_*t*_ = *b*_*i*_, *a*_*ht*_ = *a*_*h*,*j*_, *a*_*ft*_ = *a*_*f*,*k*_, *z*_*t*_ = *z*_*nz*_. Then we define the approximate forward derivative $V_{b}^{F}$ and backward derivative $V_{b}^{B}$ as follows:

$$\begin{array}{c} \begin{array}{cc} & V_{b}^{n+1}\approx \frac{V_{i+1,j,k,nz}^{n}-V_{i,j,k,nz}^{n}}{\Delta b}\approx \frac{V_{i,j,k,nz}^{n}-V_{i-1,j,k,nz}^{n}}{\Delta b}\\ & V_{b}^{F}=\frac{V_{i+1,j,k,nz}^{n}-V_{i,j,k,nz}^{n}}{\Delta b},\;V_{b}^{B}=\frac{V_{i,j,k,nz}^{n}-V_{i-1,j,k,nz}^{n}}{\Delta b} \end{array} \end{array}$$ (46)

Since $V_{b}\dot{b_{t}}=V_{b}^{B}\left(S^{c,B-}+S^{dh,B-}+S^{df,B-}\right)+V_{b}^{F}\left(S^{c,F+}+S^{dh,F+}+S^{df,F+}\right)$, we can check the sign of $\dot{b_{t}}$ and choose the approximate derivative for computation. This approximate method makes it possible for the program to solve HJB equations quickly.

Then we can get the approximate solution for the household sector by solving the discrete HJB equation. By definitions above, the discrete HJB equation is given by:

$$\begin{array}{c} \begin{array}{cc} \frac{V_{i,j,k,nz}^{n+1}-V_{i,j,k,nz}^{n}}{\Delta }+\rho V_{i,j,k,nz}^{n+1}= & \underset{c_{ht},c_{ft},l_{t}}{\text{max}\;}U+V_{b}\dot{b_{t}}+V_{ah}\dot{a_{ht}}+V_{af}\dot{a_{ft}}+V_{z}g_{z}+V_{zz}\frac{\sigma_{z}^{2}}{2}\\ = & U+V_{b}^{B}\left(S^{c,B-}+S^{dh,B-}+S^{df,B-}\right)\\ & +V_{b}^{F}\left(S^{c,F+}+S^{dh,F+}+S^{df,F+}\right)\\ & +V_{ah}^{B}d_{t}^{h,-}+V_{ah}^{F}\left(d_{t}^{h,+}+r_{aht}a_{ht}\right)+V_{af}^{B}d_{t}^{f,-}\frac{P_{t}^{*}e_{t}}{P_{t}}\\ & +V_{af}^{F}\left(d_{t}^{f,+}\frac{P_{t}^{*}e_{t}}{P_{t}}+r_{af}a_{ft}\right)\\ & +V_{z}g_{z}+V_{zz}\frac{\sigma_{z}^{2}}{2} \end{array} \end{array}$$ (47)

Note that approximate derivatives, $V_{b}^{F}=\frac{V_{i+1,j,k,nz}-V_{i,j,k,nz}}{\Delta b}$ and $V_{b}^{B}=\frac{V_{i,j,k,nz}-V_{i-1,j,k,nz}}{\Delta b}$, can be factorized. Then we rewrite the discrete HJB equation as below:

$$\begin{array}{c} \begin{array}{cc} & \frac{V_{i,j,k,nz}^{n+1}-V_{i,j,k,nz}^{n}}{\Delta }+\rho V_{i,j,k,nz}^{n+1}\\ =\; & U+\frac{V_{i-1,j,k,nz}}{\Delta b}\left(-S^{c,B-}-S^{dh,B-}-S^{df,B-}\right)\\ & +\frac{V_{i+1,j,k,nz}}{\Delta b}\left(S^{c,F+}+S^{dh,F+}+S^{df,F+}\right)\\ & +\frac{V_{i,j,k,nz}}{\Delta b}(S^{c,B-}-S^{c,F+}+S^{dh,B-}-S^{dh,F+}\\ & +S^{df,B-}-S^{df,F+})\\ & +\frac{V_{i,j-1,k,nz}}{\Delta a}\left(-d_{t}^{h-}\right)+\frac{V_{i,j+1,k,nz}}{\Delta a}\left(d_{t}^{h+}+r_{aht}a_{ht}\right)\\ & +\frac{V_{i,j,k,nz}}{\Delta a}\left(d_{t}^{h-}-d_{t}^{h+}-r_{aht}a_{ht}\right)\\ & +\frac{V_{i,j,k-1,nz}}{\Delta a^{*}}\left(-d_{t}^{f,-}\frac{P_{t}^{*}e_{t}}{P_{t}}\right)+\frac{V_{i,j,k+1,nz}}{\Delta a^{*}}\left(d_{t}^{f,+}\frac{P_{t}^{*}e_{t}}{P_{t}}+r_{aft}a_{ft}\right)\\ & +\frac{V_{i,j,k,nz}}{\Delta a^{*}}\left(d_{t}^{f,-}\frac{P_{t}^{*}e_{t}}{P_{t}}-d_{t}^{f,+}\frac{P_{t}^{*}e_{t}}{P_{t}}-r_{aft}a_{ft}\right)\\ & +\sum_{nz^{\prime }\neq nz}\lambda_{nz}\left(V_{i,j,k,nz^{\prime }}-V_{i,j,k,nz}\right), \end{array} \end{array}$$ (48)

which means *V*_*i*−1,*j*,*k*,*nz*_ and *V*_*i*+1,*j*,*k*,*nz*_ items are on different subdiagons of matrix V, *V*_*i*,*j*,*k*,*nz*_ items are on the diagonal of V. According to Achdou, et al. [21], there are same conclusions on other derivatives, so the discrete HJB equation above can be summarized as:

$$\begin{array}{c} \frac{1}{\Delta }(V_{i,j,k,nz}^{n+1}-V_{i,j,k,nz}^{n})+\rho V_{i,j,k,nz}^{n+1}=U_{i,j,k,nz}^{n}+(A^{n}+\Lambda )V_{i,j,k,nz}^{n+1}, \end{array}$$ (49)

which means that, we can get the result of period *n* + 1 if we have the value at the period *n*. By iterating this equation until the value function *V* is converged, we could solve the distribution $g\left(b_{t},a_{ht},a_{ft},z_{t}\right)≜g_{i,j,k,nz}$ for the households through the Kolmogorov Forward equation:

$$\begin{array}{c} {\left(A^{n}+\Lambda \right)}^{T}g=0 \end{array}$$ (50)

Introducing the Pollution Method in the Achdou et al. [21], as the transaction matrix *A* = (**A**^*n*^ + **Λ**)^*T*^ is a singular matrix, we randomly choose a position (*k*, *k*), let *A*[*k*, *k*] = 1, and set the *k*’s value on a zero vector as one: *ε*[*k*] = 0.01. Then we have the distribution matrix *g* ≈ *A*^−1^*ε*. According to Achdou et al. [21] and Kaplan et al. [6], the error accuracy of g is close to 10^−8^. Then we can compute aggregated values of endogenous variables by the distribution function.

### 10.2 Transaction cost

The concrete forms of transaction cost *χ*_*h*_ and *χ*_*f*_ are as follows,

$$\begin{array}{c} \begin{array}{cc} & \chi_{h}\left(d_{ht},a_{ht}\right)=\chi_{h0}\left|d_{ht}\right|+\chi_{h1}{\left|\frac{d_{ht}}{\text{max}\{a_{ht},\underline{a_{h}}\}}\right|}^{2}\text{max}\;\left\{a_{ht},\underline{a_{h}}\right\}\\ & \chi_{f}\left(d_{ft},a_{ft}\right)=Fixcost_{f}+\chi_{f0}|d_{ft}\frac{P_{t}^{*}e_{t}}{P_{t}}|+\chi_{f1}{\left|\frac{d_{ft}\frac{P_{t}^{*}e_{t}}{P_{t}}}{\text{max}\{a_{ft},\underline{a_{f}}\}}\right|}^{2}\text{max}\;\left\{a_{ft},\underline{a_{f}}\right\}\\ & \chi_{h0}\;\chi_{f0}>0,\chi_{h1}\;\chi_{f1}>1,\underline{a_{h}}\;\underline{a_{f}}>0 \end{array} \end{array}$$ (51)

Where *d*_*h*_ and *d*_*f*_ are deposits of home and foreign illiquid assets, respectively. *χ*_*h*0_ and *χ*_*h*1_ are the parameters holding home illiquid assets, and *χ*_*f*0_ and *χ*_*f*1_ are the parameters holding foreign assets. $\underline{a_{h}}$ and $\underline{a_{f}}$ are the investment thresholds for illiquid assets.

The transaction costs’ forms follow the Kaplan et al. [6], *χ*_0_|*d*_*ht*_| and $\chi_{0}^{*}|d_{ft}\frac{P_{t}}{P_{t}^{*}e_{t}}|$ are the friction cost, similar to the menu cost, going to the bank. Fixcost* is the extra costs transacting in the open economy. The quadratic term, $\chi_{h1}{\left|\frac{d_{ht}}{\text{max}\{a_{ht},\underline{a_{h}}\}}\right|}^{2}$, is caused by the home illiquid assets’ stickiness. The other quadratic term, $\chi_{f1}{\left|\frac{d_{ft}\frac{P_{t}}{P_{t}^{*}e_{t}}}{\text{max}\{a_{ft},\underline{a_{f}}\}}\right|}^{2}\text{max}\;\left\{a_{ft},\underline{a_{f}}\right\}$, is caused by the foreign illiquid assets’ stickiness.

### 10.3 Derivation for the NKPC

#### 10.3.1 Intermediate firms’ optimizations

The intermediate firms’ optimization problem is:

$$\begin{array}{c} \begin{array}{cc} \text{min}\;\; & \Pi_{j,t}=\frac{p_{j,t}}{P_{h,t}}y_{j,t}-l_{j,t}w_{t}-k_{j,t}r_{k,t}\\ st:\; & y_{i,t}=Z_{t}k_{j,t}^{\alpha }l_{j,t}^{\left(1-\alpha \right)} \end{array} \end{array}$$ (52)

Then we have the marginal cost of the intermediate firm, capital return rate and wage

$$\begin{array}{c} \begin{array}{cc} mc_{t} & =\frac{1}{Z_{t}}{\left(\frac{r_{k,t}}{\alpha }\right)}^{\alpha }{\left(\frac{w_{t}}{1-\alpha }\right)}^{1-\alpha }\\ r_{k,t} & =\alpha {\left(\frac{k_{j,t}}{l_{j,t}}\right)}^{\alpha -1}\\ w_{t} & =\left(1-\alpha \right){\left(\frac{k_{j,t}}{l_{j,t}}\right)}^{\alpha } \end{array} \end{array}$$ (53)

#### 10.3.2 NKPC

The intermediate firm *j* maximizes its profit *Π*_*j*,*t*_(*p*_*j*,*t*_) by adjusting its price *p*_*j*,*t*_ with the Rotemberg pricing adjust cost:

$$\begin{array}{c} \begin{array}{cc} \Pi_{j,t}\left(p_{j,t}\right) & =(\frac{p_{j,t}}{P_{ht}}-mc_{t}){\left(\frac{p_{j,t}}{P_{ht}}\right)}^{-\varepsilon }Y_{t}-\frac{\theta }{2}\pi_{j,t}^{2}Y_{t}\\ s.t. & \;\dot{p_{j,t}}=p_{j,t}\pi_{j,t}\\ & \;\dot{t}=1 \end{array} \end{array}$$ (54)

According to the Lemma 1 in Kaplan et al. [6], we rewrite the problem and have the HJB equation, the firm’s value function is *J*(*p*_*j*,*t*_, *t*):

$$\begin{array}{c} r_{t}^{a}J\left(p_{j,t},t\right)=\underset{\pi }{\text{max}\;}(\frac{p_{j,t}}{P_{ht}}-mc_{t}){\left(\frac{p_{j,t}}{P_{ht}}\right)}^{-\varepsilon }Y_{t}-\frac{\theta }{2}\pi_{j,t}^{2}Y_{t}+J_{p}\left(p_{j,t},t\right)p_{j,t}\pi_{j,t}+J_{t}\left(p_{j,t},t\right) \end{array}$$ (55)

The first order conditions are (simply, here omit the subscripts *j*, *t*):

$$\begin{array}{c} \begin{array}{cc} J_{p}\left(p,t\right)p & =\theta \pi Y_{t}\\ r^{a}J_{p}\left(p,t\right) & =-(\frac{p}{P_{ht}}-mc_{t})\varepsilon {\left(\frac{p}{P_{ht}}\right)}^{-\varepsilon -1}\frac{Y_{t}}{P_{ht}}+{\left(\frac{p}{P_{ht}}\right)}^{-\varepsilon }\frac{Y_{t}}{P_{ht}}\\ & +\pi J_{p}\left(p,t\right)+J_{pp}\left(p,t\right)p\pi +J_{tp}\left(p,t\right) \end{array} \end{array}$$ (56)

According to Kaplan et al. [6], in a symmetric equilibrium, *p* = *P*_*ht*_ and *y*_*j*,*t*_ = *Y*_*t*_. Thus we have:

$$\begin{array}{c} \begin{array}{cc} J_{p}\left(p,t\right) & =\frac{\theta \pi Y_{t}}{p}\\ (r^{a}-\pi )J_{p}\left(p,t\right) & =-\left(1-mc_{t}\right)\varepsilon \frac{Y_{t}}{p}+\frac{Y_{t}}{p}+J_{pp}\left(p,t\right)p\pi +J_{tp}\left(p,t\right) \end{array} \end{array}$$ (57)

Differentiating *J*_*p*_(*p*, *t*) with respect to time:

$$\begin{array}{c} J_{pp}\left(p,t\right)\dot{p}+J_{pt}\left(p,t\right)=\frac{\theta Y_{t}\dot{\pi }}{p}+\frac{\theta \dot{Y_{t}}\pi }{p}-\frac{\theta Y_{t}\pi }{p}\frac{\dot{p}}{p} \end{array}$$

Substituting *J*_*pp*_(*p*, *t*) and *J*_*p*_(*p*, *t*) intothe first order condition above, then we have:

$$\begin{array}{c} (r^{a}-\pi )\frac{\theta \pi Y_{t}}{p}=-\left(1-mc_{t}\right)\varepsilon \frac{Y_{t}}{p}+\frac{Y_{t}}{p}+\frac{\theta Y_{t}\dot{\pi }}{p}+\frac{\theta \dot{Y_{t}}\pi }{p}-\frac{\theta Y_{t}\pi^{2}}{p} \end{array}$$ (58)

Rearanging the equation above:

$$\begin{array}{c} (r^{a}-\frac{\dot{Y_{t}}}{Y_{t}})\pi =\frac{1}{\theta }\left(-\left(1-mc_{t}\right)\varepsilon +1\right)+\dot{\pi }. \end{array}$$ (59)

This is the Kew Keynesian Phillips Curve shown in KMV 2018.

### 10.4 Capital return rates

From Kaplan et al. [6], the net profits is given by:

$$\begin{array}{c} \Pi_{t}=(1-mc_{t}-\frac{\theta }{2}{\left(\frac{{\dot{P}}_{ht}}{P_{ht}}\right)}^{2})Y_{t}, \end{array}$$ (60)

thus, we have the return rate of total capital *r*_*ah*_, which equals sum of interest rate after-depreciation *r*_*k*_ − *δ* and the net profits dividends $\frac{\Pi_{t}}{K_{t}}$,

$$\begin{array}{c} r_{ah}=r_{k}-\delta +\frac{\Pi_{t}}{K_{t}}. \end{array}$$ (61)

## Supporting information

S1 Data This figure shows the import data of China and United States, 2016–2020. Trade data source: World Integrated Trade Solution, World Bank, https://wits.worldbank.org/. All data is contained in the sheet “Fig 12”.
(XLSX)

S2 Data Trade data source: World Integrated Trade Solution, World Bank, https://wits.worldbank.org/. China and US trade data is in the sheet “CN US”.
(XLSX)

S3 Data GDP grow data source: https://www.kylc.com/stats/global/yearly. GDP growth data is in the sheet “GDP growth”.
(XLSX)

S4 Data This figure shows the import data between Vietnam, China and United States, 2016–2020. Trade data source: World Integrated Trade Solution, World Bank, https://wits.worldbank.org/. All data is contained in the sheet “Fig 14”.
(XLSX)

S5 Data Trade data source: World Integrated Trade Solution—World Bank, https://wits.worldbank.org/. Vietnam and US trade data is in the sheet “VN US”.
(XLSX)
